# Symmetry‐Breaking Strategy Yields Dopant‐Free Small Molecule Hole Transport Materials for Inorganic Perovskite Solar Cells with 20.58% Efficiency and Outstanding Stability

**DOI:** 10.1002/anie.202502478

**Published:** 2025-04-11

**Authors:** Huimin Cai, Qiliang Zhu, Tianchen Pan, Lunbi Wu, Xin Gu, Chenghao Duan, Liangbin Xiong, Jiaying Wu, Sha Liu, Liyang Yu, Ruipeng Li, Keyou Yan, Ruijie Ma, Shengjian Liu, Tao Jia, Gang Li

**Affiliations:** ^1^ School of Optoelectronic Engineering Guangdong Polytechnic Normal University Guangzhou 510665 P.R. China; ^2^ School of Environment and Energy State Key Laboratory of Luminescent Materials and Devices Guangdong Provincial Key Laboratory of Solid Wastes Pollution Control and Recycling South China University of Technology Guangzhou 510000 P.R. China; ^3^ Advanced Materials Thrust Function Hub The Hong Kong University of Science and Technology (Guangzhou) Nansha Guangzhou 511400 P.R. China; ^4^ Longzihu New Energy Laboratory School of Energy Science and Technology Henan University Zhengzhou 450000 P.R. China; ^5^ Dongguan Key Laboratory of Interdisciplinary Science for Advanced Materials and Large‐Scale Scientific Facilities School of Physical Sciences Great Bay University Dongguan Guangdong 523000 P.R. China; ^6^ Research Institute of Frontier Science Southwest Jiaotong University Chengdu 610031 P.R. China; ^7^ School of Chemical Engineering Sichuan University Chengdu 610065 P.R. China; ^8^ National Synchrotron Light Source (NSLS II) Brookhaven National Laboratory Upton NY 11973 USA; ^9^ Department of Electrical and Electronic Engineering Research Institute for Smart Energy (RISE), Photonic Research Institute (PRI), Guangdong‐Hong Kong‐Macao Joint Laboratory for Photonic‐Thermal‐Electrical Energy Materials and Devices The Hong Kong Polytechnic University Hong Kong P.R. China; ^10^ School of Chemistry Guangzhou Key Laboratory of Materials for Energy Conversion and Storage Key Laboratory of Electronic Chemicals for Integrated Circuit Packaging South China Normal University (SCNU) Guangzhou 510006 P.R. China

**Keywords:** Dopant‐free, Hole transport material, Inorganic perovskite solar cell, Small molecule, Symmetry‐breaking

## Abstract

Inorganic perovskites are known for their excellent photothermal stability; however, the photothermal stability of all‐inorganic n‐i‐p perovskite solar cells (PSCs) is compromised due to ion diffusion and free radical‐induced degradation caused by the use of doped spiro‐OMeTAD hole transport materials (HTMs). In this study, two isomeric donor–acceptor–donor (D–A–D) type small molecules, namely HBT and HiBT, were developed and used as dopant‐free HTMs, using 2,1,3‐benzothiadiazole or benzo[*d*][1,2,3]thiadiazole as acceptor moieties. The HiBT molecule, with its symmetry‐breaking features, exhibits a large dipole moment, enhanced coordination‐active sites, and a well‐aligned energy level structure, all of which contribute to passivating perovskite surface defects and improving free charge separation. As a result, inorganic CsPbI_3_ PSCs with HiBT HTM achieved an impressive power conversion efficiency (PCE) of 20.58%, the highest reported for dopant‐free HTM‐based inorganic PSCs. Moreover, the enhanced hydrophobic properties of HiBT molecules, coupled with their ability to passivate perovskite surface defects, contribute to significantly improved device stability. The unencapsulated devices based on HiBT HTM retained over 83% and 80% of their initial efficiency after being stored at 85 °C for 50 days and undergoing maximum power point (MPP) tracking at 85 °C for 1100 h, respectively. These results highlight that the symmetry‐breaking strategy is an exceptionally effective approach for designing efficient, dopant‐free small molecule HTMs, significantly contributing to both the high efficiency and enhanced stability of all‐inorganic PSCs.

## Graphical abstract

A symmetry‐breaking strategy was used to design two isomeric D–A–D small molecules with 2,1,3‐benzothiadiazole or benzo[*d*][1,2,3]thiadiazole as acceptor motifs for dopant‐free hole transport materials in CsPbI₃ perovskite solar cells. Structural asymmetry can effectively regulates the dipole moment, energy level alignment, coordination‐active sites, and thereby enabling a record PCE of 20.58% with outstanding stability.

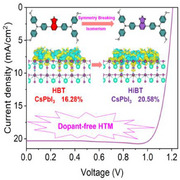

## Introduction

Over the past decade, the power conversion efficiency (PCE) of hybrid organic–inorganic perovskite solar cells (PSCs) has surged, exceeding 27%, underscoring their tremendous potential as a low‐cost, next‐generation photovoltaic technology.^[^ ^1^, ^2^, ^3^, ^4^, ^5^ ^]^ However, the inherent instability of organic components, such as methylammonium (MA⁺) or formamidinium (FA⁺), under thermal and light stress poses a significant obstacle to commercialization.^[^ ^6^, ^7^, ^8^ ^]^ Inorganic perovskites, such as CsPbI_3_, in which organic cations are replaced with cesium (Cs⁺), exhibit superior photothermal stability and have consequently attracted significant attention.^[^ ^9^, ^10^, ^11^, ^12^ ^]^ Currently, the state‐of‐the‐art n‐i‐p CsPbI_3_ PSCs primarily rely on 2,2′,7,7′‐tetrakis(*N,N*‐di‐methoxyphenylamine)‐9,9′‐spirobifluorene (spiro‐OMeTAD) as the hole transport material (HTM).^[^ ^13^, ^14^, ^15^ ^]^ To enhance PSC performance, spiro‐OMeTAD has to be doped, dominantly with bis(trifluoromethane)sulfonimide lithium salt (LiTFSI) and 4‐*tert*‐butylpyridine (tBP).^[^ ^16^, ^17^ ^]^ However, these dopants compromise the long‐term stability of the device due to issues such as ionic diffusion and degradation induced by free radicals.^[^ ^18^, ^19^, ^20^ ^]^ Additionally, the hygroscopic nature of the dopants during oxidation in air accelerates the decomposition of the CsPbI_3_ film, further exacerbating stability challenges.^[^ ^21^, ^22^ ^]^ To overcome these limitations, the development of dopant‐free, efficient, and stable HTMs for inorganic PSCs has emerged as a critical research focus for enhancing both the performance and durability of PSCs, paving the way for their commercialization.^[^ ^23^, ^24^, ^25^ ^]^

**Figure 1:**
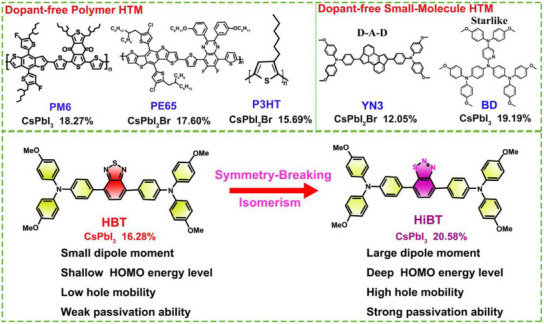
a) Representative dopant‐free HTMs and their performances for inorganic PSCs. b) The molecular structures of HBT and HiBT HTMs in this work.

Dopant‐free HTMs applied to inorganic PSCs can be broadly categorized into polymers and small molecules (Figure 1a). Polymer HTMs generally deliver higher efficiency in inorganic PSCs. For instance, Song et al. achieved a power conversion efficiency (PCE) of 18.27% using the dopant‐free polymer HTM PM6 in CsPbI_3_ PSCs.^[^ ^26^ ^]^ Similarly, Zhou et al. demonstrated that substituting spiro‐OMeTAD with the DTBDT‐based polymer HTM (PE65) in CsPbI₂Br PSCs resulted in a PCE of 17.60%.^[^ ^27^ ^]^ However, from an industrial perspective, polymer‐based HTMs have shortcomings including their inherent high synthetic complexity, low reproducibility, and high material costs. In contrast, small molecule dopant‐free HTMs offer advantages such as well‐defined structure, ease of synthesis, structural tunability, and straightforward purification. For example, Zheng et al. reported a PCE of 12.41% for CsPbI₂Br PSCs using a small molecule donor–acceptor–donor (D–A–D) linear HTM without doping.^[^ ^28^ ^]^ In our previous work, we demonstrated the application of a star‐shaped dopant‐free BD HTM with twisted acceptor units and strong dipoles in CsPbI_3_ PSCs, achieving a record‐breaking efficiency of 19.19%.^[^ ^29^ ^]^ However, small molecule HTM‐based inorganic PSCs often exhibit lower efficiency compared to those using polymer HTMs. Therefore, it is very meaningful to develop novel, low‐cost, dopant‐free small molecule HTMs to achieve highly efficient and stable all‐inorganic PSCs.

Here, we present two small‐molecule HTMs by focusing on the isomerization acceptor building blocks. The molecules feature a linear D–A–D molecular backbone composed of benzo[*c*][1,2,5]thiadiazole or benzo[*d*][1,2,3]thiadiazole acceptor units bridging triphenylamine (TPA) moieties symmetrically on both ends, named HBT and HiBT (Figure 1b). The symmetry breaking induced by the benzo[*d*][1,2,3]thiadiazole unit allows for precise regulation of the dipole moment, energy levels, hole mobility, and intermolecular order. Meanwhile, isomerization enhances the interaction with uncoordinated Pb^2+^, helping to passivate defects on the perovskite film surface and reduce interface charge losses. As a result, the HiBT‐based CsPbI_3_ PSC achieved an optimal efficiency of 20.58%, representing the highest efficiency reported for inorganic PSCs utilizing dopant‐free HTMs. Additionally, the predominant passivation capability and amorphous nature of HiBT contribute to the enhanced stability of the related PSCs. Compared to the rapid efficiency roll‐off observed in devices using doped spiro‐OMeTAD, the unencapsulated HiBT‐based PSCs demonstrated exceptional long‐term stability, retaining 83% of the initial PCE after 50 days of storage at 85 °C, and approximately 80% of the initial efficiency after 1100 h of maximum power point (MPP) tracking at 85 °C. This work demonstrates that the symmetry‐breaking molecular design strategy is an effective approach for developing highly efficient dopant‐free small molecule HTMs, leading to high‐efficiency and highly stable inorganic PSCs.

## Results and Discussion

### Molecular Design of Small‐Molecule HTMs

The newly developed HTMs feature a D–A–D linear conjugated molecular backbone, which was commonly utilized in the design of dopant‐free HTMs. Two key moieties are involved in the design of dopant‐free HTMs: TPA, renowned for its electron‐rich nature and crucial role in charge transport, serves as the ideal donor (D) unit, while 2,1,3‐benzothiadiazole or benzo[*d*][1,2,3]thiadiazole serves as acceptor to construct the molecular backbone (Figure 1b). Changing from 2,1,3‐benzothiadiazole to benzo[*d*][1,2,3]thiadiazole introduces symmetry‐breaking properties and aims to increase the dipole moment, improve luminescence efficiency, and fine‐tune energy level structure, thereby facilitating efficient charge extraction and suppressing nonradiative recombination to achieve higher open‐circuit voltage (*V*_OC_). The detailed synthetic routes for HBT and HiBT are provided in the supporting materials. In brief, both HTMs are synthesized from inexpensive raw materials through a simple one‐step process with ∼90% yields. The structures of HBT and HiBT were confirmed via nuclear magnetic resonance (NMR) spectroscopy and mass spectrometry (Figures S1–S6, Supporting Information). Considering the importance of material cost for the commercialization of PSCs, we estimated the production costs of HiBT and HBT to be approximately 10.16 and 10.52 dollars g^−1^, respectively. These costs are primarily driven by the low price of the BriBT and BrBT unit (Table S1, Supporting Information), which is significantly lower than that of the commercial spiro‐OMeTAD (118.19–611.79 dollars/g^−1^).

### Theoretical Calculations and Basic Properties of HTMs

**Figure 2:**
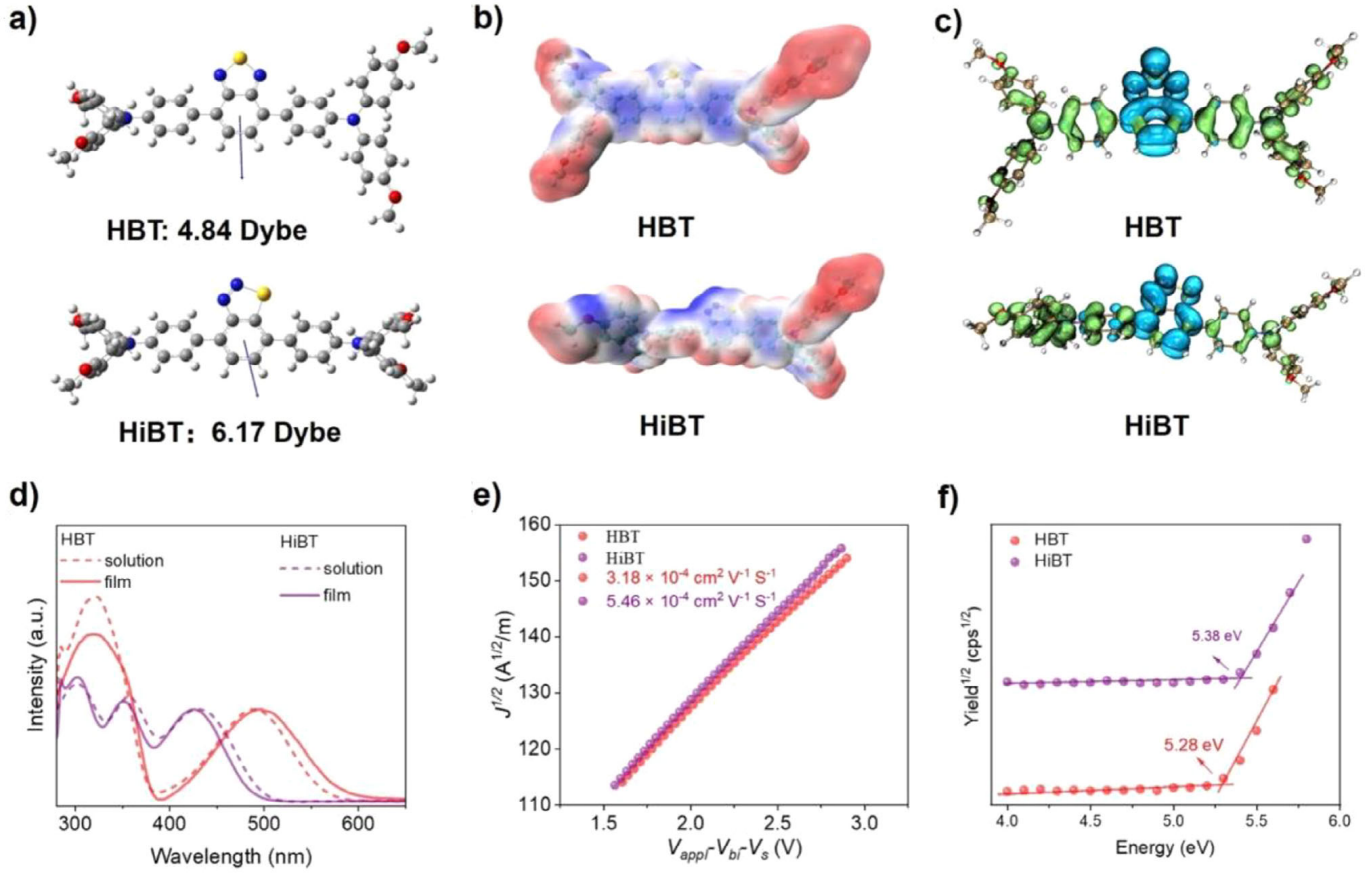
a) Dipole moments of HBT and HiBT. d) ESP distributions of HBT and HiBT. c) The electron‐hole distributions of HBT and HiBT. d) Absorption spectra of HBT and HiBT. e) Hole mobilities of HBT and HiBT measured by SCLC method. f) PESA measurements and the ionization potentials of HBT and HiBT.

The geometric configurations and electronic properties of the newly developed HTMs were investigated using density functional theory (DFT) calculations. Due to its highly symmetrical structure, HBT exhibits a slightly lower dipole moment of 4.84 D (Figure 2a). Meanwhile, by breaking the symmetry, HiBT concentrates nitrogen atoms on one side, disrupting the original symmetry and resulting in a large dipole moment of 6.17 D. The increased dipole moment potentially enhances the interaction between the perovskite and the HTM, thereby facilitating the separation and extraction of free charges.^[^ ^30^, ^31^, ^32^, ^33^ ^]^ As depicted in Figure 2b, the HBT molecule exhibits an axisymmetric electrostatic potential (ESP) distribution. In contrast, the isomer HiBT displays an asymmetric ESP distribution, with a more negative potential near the azo group and a relatively neutral distribution at the sulfur atom position. This result was consistent with the atomic charge analyses (Figure S8). Notably, the nitrogen atom carries a negative charge, suggesting that the lone pair electrons on the nitrogen atom can potentially passivate defects on the perovskite surface through Lewis acid–base interactions.^[^ ^34^, ^35^ ^]^ Moreover, the symmetry‐breaking strategy can endow HiBT with a deeper highest occupied molecular orbital (HOMO) level, which is conducive to obtaining higher *V*_OC_ in PSC applications (Figure S9).^[^ ^36^ ^]^ The hole‐electron distributions of HBT and HiBT are shown in Figure 2c. The electron distribution in HBT is more centralized, while in HiBT, it shifts towards the azo group. Furthermore, the HiBT molecule demonstrates enhanced charge separation, which strengthens electron transport process and potentially improves its interaction with the perovskite layer.^[^ ^37^, ^38^ ^]^ The charge density differences of HTMs/perovskite structures were investigated to address the defect passivation effect from molecular point of view. As shown in Figure S10, the total electron density differences reveal the interactions between HTMs and perovskite.^[^ ^39^ ^]^ Although both systems exhibit uniformly distributed electron density differences at the HTM–perovskite interfaces and the presence of multiple nitrogen (N), oxygen (O), and sulfur (S) atoms in HTMs enhances these interfacial interactions, HiBT indeed demonstrates stronger charge transfer between its nitrogen atoms and perovskite due to symmetry breaking in its molecular structure, which verifies the more effective passivation with undercoordinated Pb^2^⁺ ions at the interface. This enhanced defect passivation capability helps reduce nonradiative recombination in devices, thereby improving the photovoltaic parameters.^[^ ^40^, ^41^ ^]^ Another key consideration for HTMs in perovskite applications is their stability. In general, doped spiro‐OMeTAD undergoes a phase transition from amorphous to crystalline under high‐temperature conditions, which negatively impacts the device stability.^[^ ^42^ ^]^ To assess the thermal stability of HBT and HiBT, thermogravimetric and differential scanning calorimetric analyses were conducted. As shown in Figure S11, both HBT and HiBT exhibit excellent thermal stability, with thermal decomposition temperatures (*T*_d_) at 5% weight loss of 324 and 382 °C, respectively. These results suggest that HBT and HiBT have the potential to serve as stable HTMs in PSCs. A high glass transition temperature (*T*_g_) is essential for maintaining the stable morphology of HTMs. As shown in Figure S12, the *T*_g_ values of the synthesized HBT and HiBT HTMs are 94 and 96 °C, respectively, which was significantly higher than that of doping spiro‐OMeTAD (40 °C), ensuring better stability of the PSCs.^[^ ^43^, ^44^, ^45^ ^]^ The absence of melting peak across the entire tested range confirms the amorphous nature of both molecules, a key factor for ensuring interfacial stability. In addition, both HBT and HiBT are soluble in chlorobenzene, allowing for the formation of hole transport layers (HTLs) through solution processing.

The absorption spectra of HBT and HiBT in both solid state and chlorobenzene solution were investigated (Figure 2d). The absorption spectra reveal that both small molecules exhibit multipeak absorption characteristics. The absorption below 400 nm is attributed to π–π* transitions, while the absorption above 400 nm corresponds to intermolecular charge transfer from the electron‐donating unit to the electron‐accepting group. From solution to film, the absorption of both molecules exhibits a redshift, indicating enhanced aggregation in the film state. Based on the onset of the absorption spectra for HBT and HiBT films, their corresponding bandgaps are estimated to be 2.13 and 2.49 eV, respectively. The hole mobilities of HBT and HiBT were investigated by the space charge limited current (SCLC) method. As shown in Figure 2e, the hole mobility of HiBT is calculated to be 5.46 × 10⁻⁴ cm^2^ V⁻¹ s⁻¹, which is significantly higher than that of HBT (3.18 × 10⁻⁴ cm^2^ V⁻¹ s⁻¹). These higher mobilities can facilitate efficient hole transport and collection without a doping process for PSCs, thereby indicating two molecules are expected to be promising candidates for the application of dopant‐free HTM in PSCs.^[^ ^46^ ^]^ The energy band structures of HBT and HiBT were analyzed by photoelectron spectroscopy in air (PESA) measurement. HiBT shows an ionization potential of 5.38 eV, which is 0.10 eV deeper than that of HBT (5.28 eV) (Figure 2f). This indicates that the energy levels of the HTMs align well with the valence band (VB) of the inorganic CsPbI_3_ perovskite, promoting efficient charge transfer and reducing *V*_OC_ losses in the device. By subtracting the optical bandgap, the LUMO energy levels of HBT and HiBT were calculated to be −3.15 and −2.89 eV, respectively. The shallower LUMO level of HiBT can help block electrons, thereby improving device performance. Overall, the characterization results above suggest that HiBT holds considerable potential as a dopant‐free HTM for high‐performance inorganic PSCs.

### Film Microstructure

**Figure 3:**
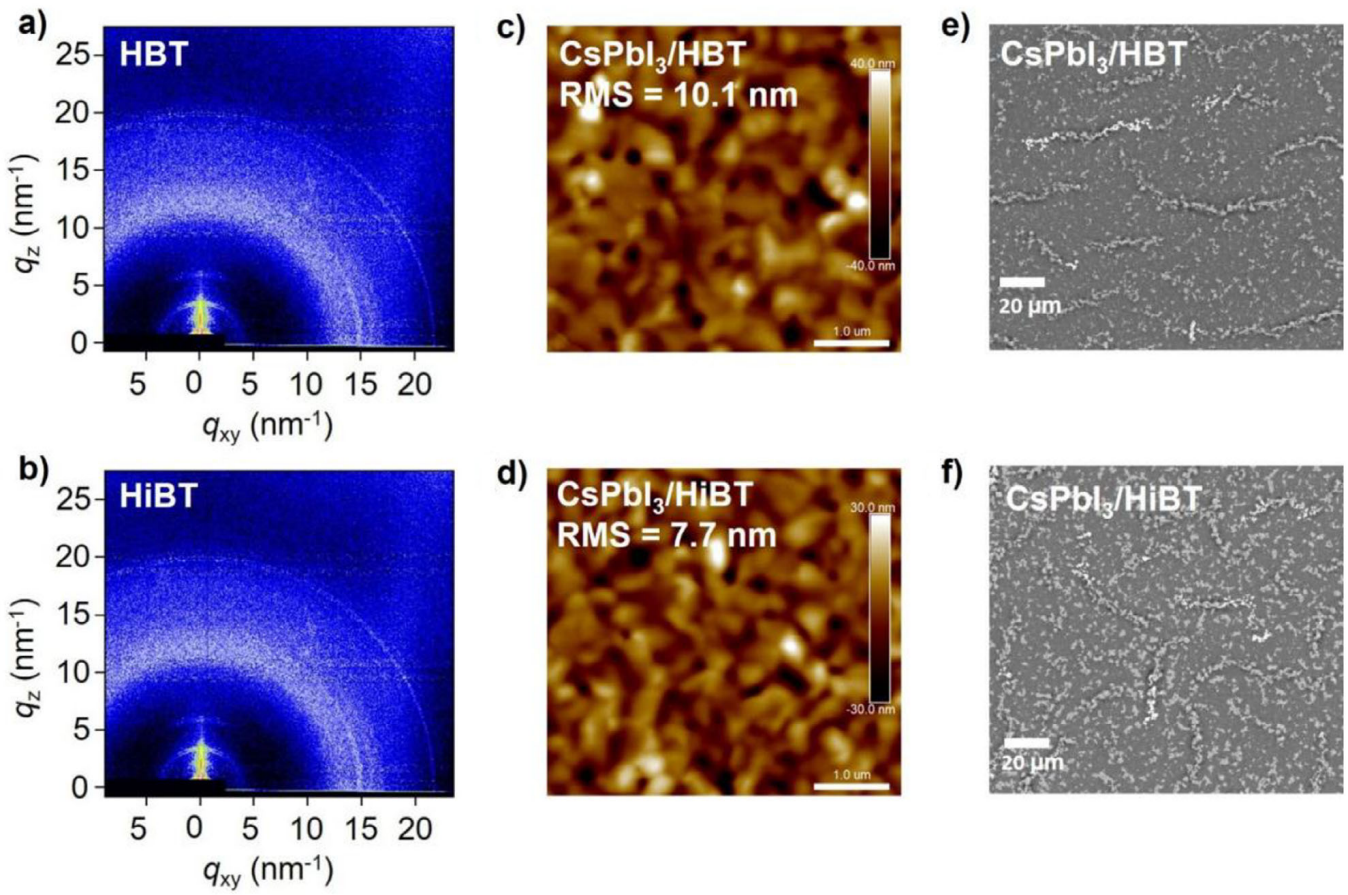
GIWAXS patterns of a) HBT and b) HiBT films. The AFM images of c) HBT and d) HiBT films deposited on CsPbI_3_ films. The SEM images of e) perovskite/HBT and f) perovskite/HiBT films.

The molecular crystallization and orientation were examined using grazing‐incidence wide‐angle X‐ray scattering (GIWAXS) measurements, which revealed that both HBT and HiBT films exhibited relatively weak crystallinity, as indicated by the broad diffraction rings or Bragg peaks (Figure 3a,b). As shown in Figure S13, both films exhibit two distinct peaks at *q* = 1.3 and *q* = 14.4 nm⁻¹, which correspond to the lamellar stacking direction and π–π stacking orientation, respectively. The intensity of these peaks in the HiBT film was slightly higher than in the HBT film, indicating that HiBT molecules are more tightly packed, which facilitates higher hole mobility.^[^ ^47^ ^]^ The slight ordered stacking of HiBT molecules helps facilitate interface contact at both the perovskite/HTM and HTM/top electrode interfaces within the device. The film morphology of the HTL plays a crucial role in determining the performance of PSCs. We investigated the deposition morphology of different HTMs on perovskite films using atomic force microscopy (AFM) and scanning electron microscopy (SEM) to assess their film‐forming properties. The root mean square (RMS) roughness of the pure CsPbI_3_ perovskite film was measured to be 16.70 nm (Figure S14). After spin coating the HTM onto the perovskite layer, the HiBT‐coated film shows an RMS value of 7.72 nm, which is smaller than the HBT‐coated film (10.10 nm) (Figure 3c,d). This is because the symmetry‐breaking structure of HiBT enhances solubility in chlorobenzene, thereby leading to a more uniform film surface. Figure 3e,f show SEM images of different HTMs deposited on the perovskite film. The HBT film exhibits relatively uneven distribution, while HiBT‐coated film reveals more uniform and complete coverage (Figures 3f and S15). This improvement in film quality facilitates the efficient transport and extraction of free‐charge carriers from the perovskite layer.

### Device Performance

**Figure 4:**
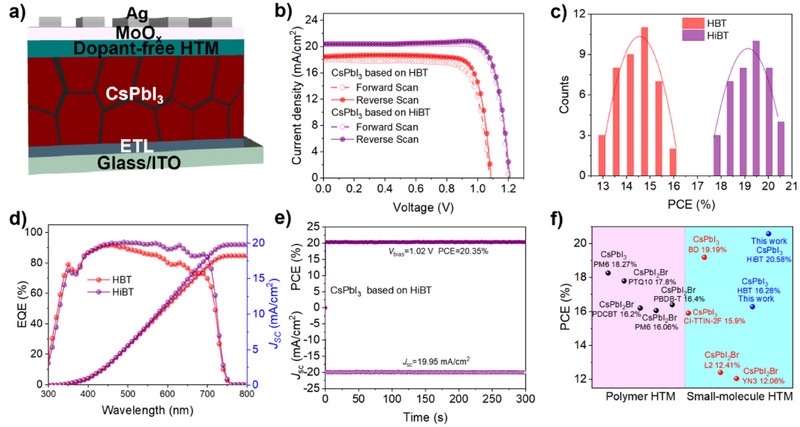
a) Schematic diagram of the CsPbI_3_ PSC structure. b) *J*–*V* characteristic curves for n‐i‐p CsPbI_3_ solar cells with HBT and HiBT HTLs. c) Histograms illustrating the PCE of CsPbI_3_ PSCs based on HBT and HiBT HTLs. d) EQE spectra of the optimal devices with HBT and HiBT HTLs. e) Steady‐state *J*_SC_ and PCE measurements of p‐i‐n CsPbI_3_ PSCs with HiBT HTL. f) Efficiency statistics of all‐inorganic PSCs utilizing dopant‐free HTMs.

To evaluate the photovoltaic performance of HBT and HiBT as dopant‐free HTMs, we fabricated n‐i‐p CsPbI_3_ PSCs with the structure ITO/SnO_2_/CsPbI_3_/HTM/MoO_3_/Ag (Figure 4a). The optimized thicknesses of the HBT and HiBT HTMs were approximately 30 and 25 nm, respectively (Figure S16). Notably, compared to doped spiro‐OMeTAD case, a lower concentration of HTM was sufficient to fully cover the perovskite layer, allowing the free charges generated by the perovskite to reach the metal electrode more efficiently through the HTL. The current density–voltage (*J*–*V*) curves of the champion devices based on HBT and HiBT are shown in Figure 4b, and the corresponding photovoltaic parameters are summarized in Table 1. The HBT‐based control device achieved a PCE of 16.28% (15.01% for the forward scan), with an open‐circuit voltage (*V*_OC_) of 1.09 V, a short‐circuit current (*J*_SC_) of 18.39 mA cm^−2^, and a fill factor (FF) of 81.39%. In contrast, the HiBT‐based devices exhibited a significantly enhanced PCE of 20.58% (19.94% for the forward scan), a *J*_SC_ of 20.33 mA cm^−2^, an FF of 83.70%, and a *V*_OC_ of 1.21 V. The superior performance of the HiBT‐based devices is primarily attributed to the synchronous increases in *V*_OC_, *J*_SC_, and FF parameters, which can be ascribed to the improved energy alignment between the perovskite and HTM, along with the passivation effect of HiBT on the perovskite. Figure 4c illustrates the performance distributions of PSC devices based on two HTMs. It is evident that the efficiency distributions for both HBT‐ and HiBT‐based devices are relatively concentrated, with the HiBT‐based devices demonstrating consistently higher overall performance. This indicates good repeatability for devices based on these two HTMs. We also examined the effect of various HTM concentrations on device performance. As shown in Figures S17, S18, and Tables S2, S3, the optimal device efficiency was achieved at concentrations of 20 mg mL⁻¹ for HBT and 15 mg mL⁻¹ for HiBT HTMs. Compared to the high‐concentration spiro‐OMeTAD HTM, the lower concentrations of HBT and HiBT not only improve the conductivity of the HTLs but also reduce material consumption, thereby lowering the overall fabrication costs of the devices.

**Table 1:** Device parameters of CsPbI_3_ PSCs with dopant‐free HBT or HiBT H.

Samples	*V*_oc_ (V)	*J*_sc_ (mA cm^−2^)	FF (%)	PCE (%)
HBT				
Forward scan	1.08	18.00	77.29	15.01
Reverse scan	1.09 (1.04 ± 0.05)^a)^	18.39 (16.71 ± 1.52)	81.39 (73.43 ± 7.87)	16.28 (14.56 ± 1.73)
HiBT				
Forward scan	1.20	20.33	81.57	19.94
Reverse scan	1.21 (1.18 ± 0.03)	20.33 (19.07 ± 1.26)	83.70 (78.92 ± 4.76)	20.58 (19.64 ± 0.95)

^a)^ Data in parentheses are the average values based on 40 independent devices.

The external quantum efficiency (EQE) spectral integral *J*_SC_ for HBT and HiBT devices was 18.19 and 19.74 mA cm^−2^, respectively (Figure 4d), which is in good agreement with the *J*_SC_ measured by *J*–*V* characteristics. Figure 4e exhibits that the HiBT device obtains a stable output efficiency of 20.35% under maximum power point (MPP) with 300 s. Finally, we summarized the performance of inorganic PSCs based on dopant‐free HTM reported so far, and the relevant statistical data are listed in Table S4. We can observe that the HiBT CsPbI_3_ device achieves the highest efficiency among all reported inorganic PSCs based on dopant‐free HTM (Figure 4f).

### Device Physical Characteristics, Passivation Effect, and Chemical Bonding

**Figure 5:**
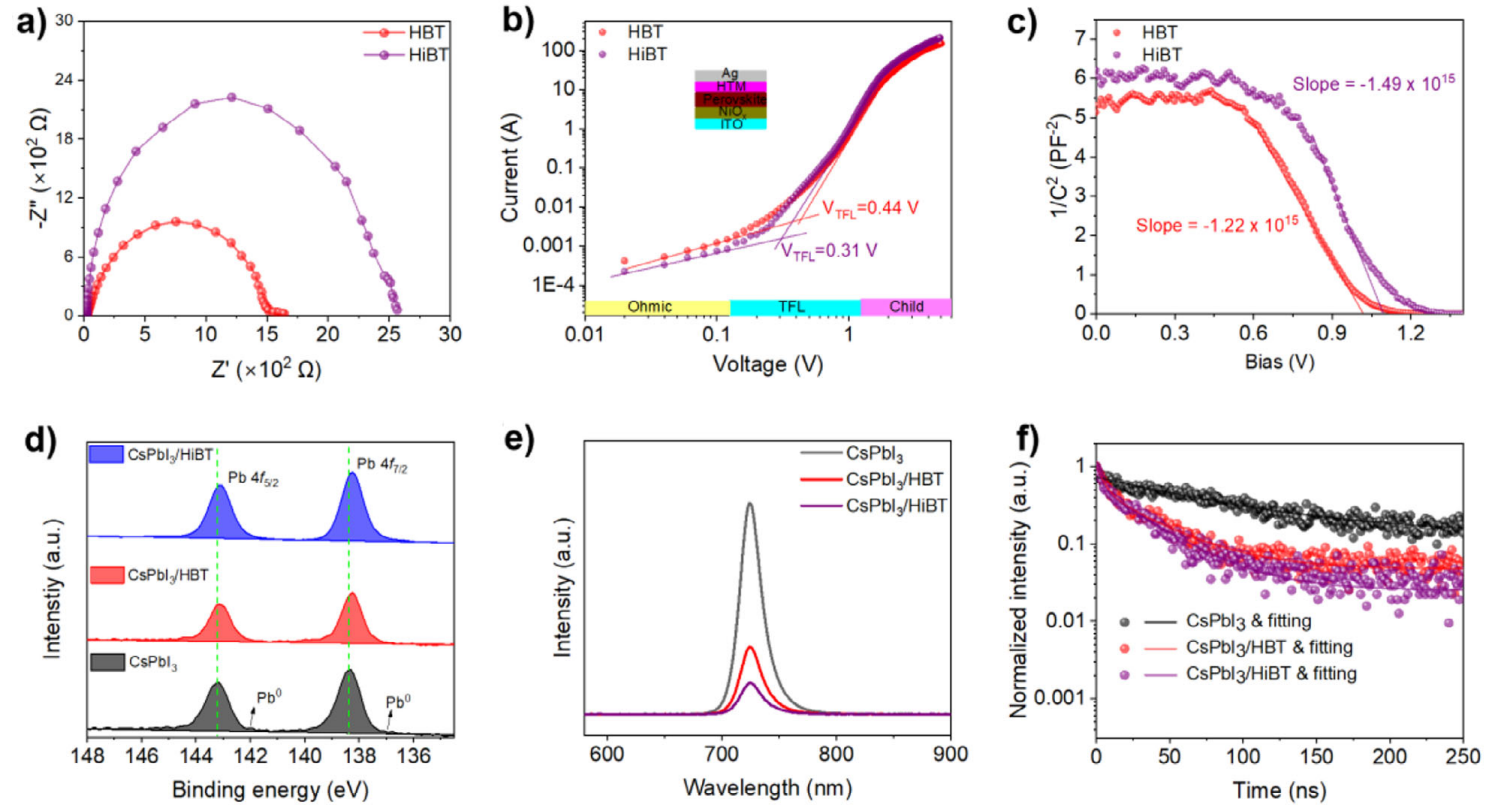
a) Nyquist plots. b) The space‐charge‐limited current versus voltage of devices (ITO/NiO*_x_*/perovskite/HTM/Ag) with HBT and HiBT HTLs. c) Mott–Schottky measurement for the CsPbI_3_ devices with HBT and HiBT HTLs. d) XPS Pb 4f spectra of CsPbI_3_, CsPbI_3_/HBT, and CsPbI_3_/HiBT films. e) Steady‐state PL and f) TRPL spectra for pristine CsPbI_3_, CsPbI_3_/HBT, and CsPbI_3_/HiBT films.

To elucidate the charge transfer and recombination dynamics of the device, electrochemical impedance spectroscopy (EIS) was employed for quantitative analysis. Figure 5a presents the Nyquist plots and corresponding equivalent circuit models for devices based on HBT and HiBT, measured under dark conditions at *V*_OC_. The HiBT‐based device exhibited a smaller series resistance (Rs) compared to the HBT‐based device, indicating enhanced charge transfer kinetics. Additionally, the use of HiBT HTM led to a significant increase in recombination resistance (*R*_rec_) from 1498 to 2560 Ω, suggesting that HiBT as HTL can effectively passivate defects and suppress charge recombination within the device.

The effects of HBT and HiBT on the trap density of CsPbI_3_ thin films were further examined through space charge‐limited current (SCLC) characterization (Figure 5b). The dark current density‐voltage curve reveals three distinct regions: the ohmic region, the trap‐filled region, and the child region. After the deposition of HBT and HiBT HTMs, the trap‐filled limit voltage (*V*_TFL_) was measured to be 0.44 V for HBT and 0.31 V for HiBT, with the corresponding trap state densities of 1.52 × 10¹⁵ and 1.07 × 10¹⁵ cm⁻^3^, respectively. These results confirm that HiBT effectively passivates uncoordinated Pb^2^⁺ defects on the surface of CsPbI_3_ perovskite through interaction with the perovskite layer.^[^ ^48^ ^]^ The perovskite/HTM interface properties were analyzed using capacitance–voltage (*C*–*V*) measurements (Figure 5c). The *C*⁻^2^–*V* curves for devices based on HiBT and HBT followed the Mott–Schottky equation (Equation 1):

(1) $$C^{-2}=\frac{\left(2V_{\mathrm{bi}}-V\right)}{A^{2}q\varepsilon_{0}\varepsilon N}$$

where *C* represents the capacitance, *V*_bi_ is the built‐in potential, *N* is the charge density, and *V* is the applied voltage. *C*, *ε*, and *ε*₀ correspond to the depletion layer capacitance, relative permittivity, and vacuum permittivity, respectively. *V*_bi_, arising from carrier diffusion, is a crucial factor for charge injection in solar cells. The estimated *V*_bi_ for the HiBT‐based device (1.09 V) exceeds that of the HBT‐based device (1.02 V), which aligns with the lower HOMO energy level of HiBT. This increase in *V*_bi_ extends the depletion zone, enhancing the driving force for carrier injection and directly contributing to the higher *V*_OC_. Furthermore, the steeper slope of the Mott–Schottky plot for HiBT‐based devices indicates a lower interfacial charge density, which improves charge extraction.^[^ ^49^ ^]^

To further investigate the potential interaction between perovskite and HTM, X‐ray photoelectron spectroscopy (XPS) measurements were performed to analyze the chemical bonding states at the perovskite/HTM interface.^[^ ^50^ ^]^ The high‐resolution Pb 4*f* spectra, shown in Figure 5d, reveal the presence of two main peaks corresponding to Pb 4f_7/2_ and Pb 4f_5/2_ peaks. In the CsPbI_3_ film, additional peaks at 137.1 and 142.0 eV were observed, indicating the presence of metallic Pb. The significant presence of metallic Pb suggests the existence of iodide vacancies or uncoordinated Pb^2+^ defects, which can act as nonradiative recombination centers, thus impairing the performance of PSCs. Compared to the pure perovskite film, the Pb 4f characteristic peak in the perovskite/HBT and perovskite/HiBT samples shifted to lower binding energies, with the metallic Pb peaks significantly suppressed. Additionally, the N 1s peaks of HBT and HiBT shifted to higher binding energies in the perovskite/HBT and perovskite/HiBT samples (Figures S19, S20). This shift is attributed to changes in the electron density around the atoms, as the N, O, S atoms can donate their lone pair electrons to the empty 6p orbital of Pb^2+^. These observations suggest that both HBT and HiBT can effectively passivate the trap states on the surface of perovskite films.^[^ ^51^ ^]^

Charge extraction properties are critical for HTMs, and we investigated the charge carrier dynamics in pristine CsPbI_3_ and CsPbI_3_/HTM films using steady‐state photoluminescence (PL) and time‐resolved photoluminescence (TRPL) spectroscopy. The pure CsPbI_3_ film exhibited the strongest PL emission peak centered at 725 nm (Figure 5e). The introduction of HTMs significantly quenched the PL, with HiBT demonstrating the lowest PL intensity, indicating superior hole extraction capability compared to HBT. TRPL spectroscopy was employed to quantitatively analyze the charge carrier dynamics (Figure 5f and Table S5). The pure CsPbI_3_ film exhibited a longer carrier lifetime (*τ* = 73.43 ns), which decreased upon the introduction of HTMs due to charge extraction. The hole transfer rate at the CsPbI_3_/HiBT film (27.96 ns) was faster than at the CsPbI_3_/HBT sample (33.01 ns), which is likely attributed to the deeper HOMO energy level, higher hole mobility of HiBT, and stronger interfacial interaction between Pb^2+^ ions on the perovskite surface and the N atom of HiBT compared to HBT.

### Device Stability

**Figure 6:**
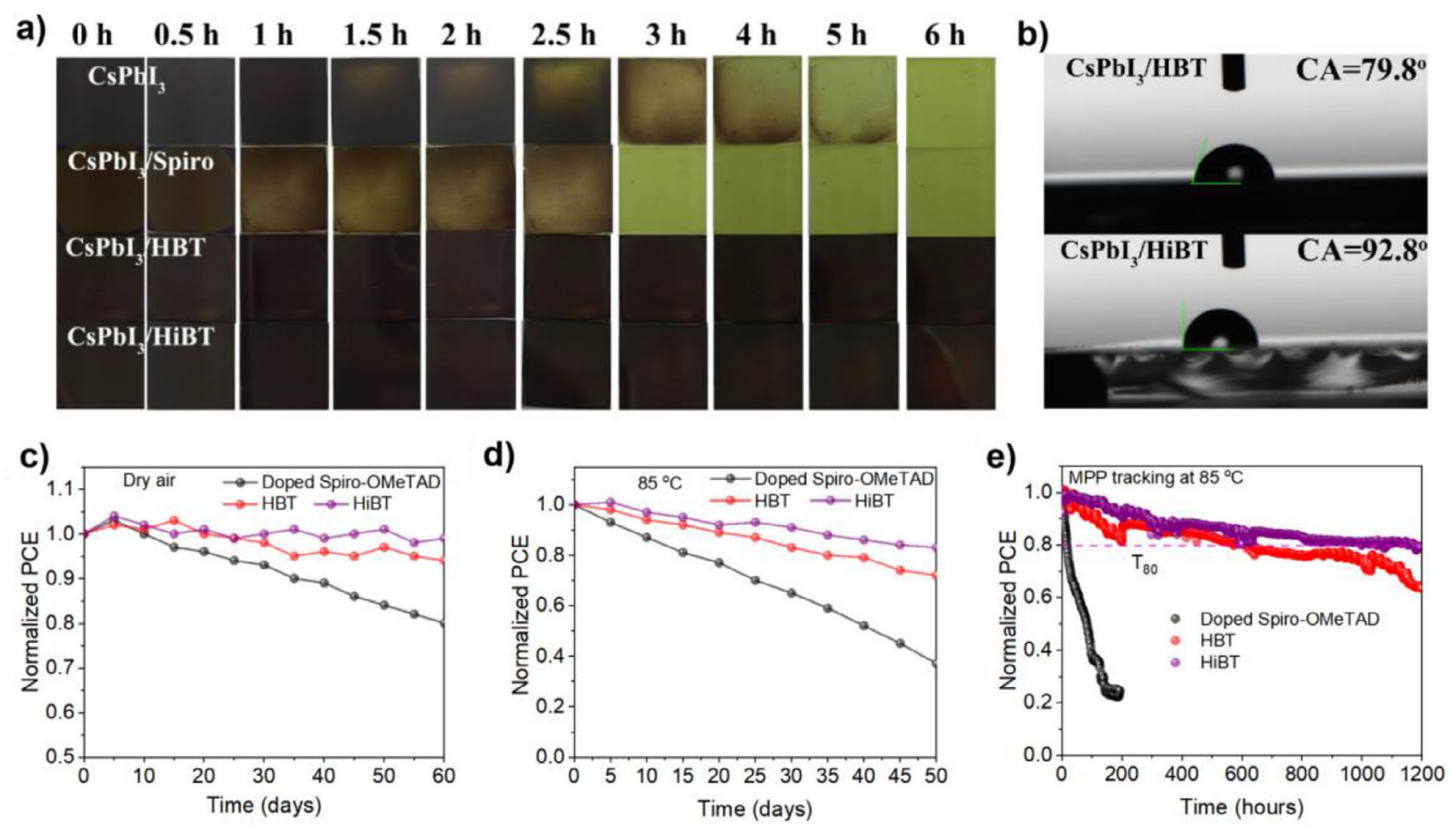
a) Photos of the CsPbI_3_ films with and without different HTLs, taken before and after 6‐h exposure to a relative humidity (RH) of 50 ± 5%. b) Contact angles of CsPbI_3_/HBT and CsPbI_3_/HiBT films. c)–e) Normalized PCE changes of the inverted devices based on doped spiro‐OMeTAD, dopant‐free HBT, and HiBT under the condition of c) dry air, d) 85 °C, and e) MPP tracking at 85 °C.

Stability is one of the most critical factors in evaluating the performance of PSCs.^[^ ^52^, ^53^ ^]^ To assess the impact of different HTMs on the long‐term stability of PSCs, we examined the stability of unencapsulated devices under various storage conditions. Moisture‐induced phase transformation of the perovskite film is a predominant degradation pathway for all‐inorganic PSCs. To investigate the effect of HTLs on the phase stability of perovskite films, we monitored the evolution of CsPbI_3_ films under controlled relative humidity (RH) = 50%. Photographic evidence of perovskite films stored over various time intervals is presented in Figure 6a. Notably, the CsPbI_3_ film coated with doped spiro‐OMeTAD exhibited bleaching within 1 h, a process significantly accelerated compared to the pure CsPbI_3_ film (1.5 h). In contrast, the CsPbI_3_ films with HBT and HiBT HTLs showed no significant color change even after 6 h of exposure.

To further investigate the long‐term phase stability of perovskite films at RH = 50%, we performed X‐ray diffraction (XRD) analysis. All freshly prepared CsPbI_3_ films exhibited the typical black phase, characterized by two prominent peaks at 14.6° and 29.2°, corresponding to the (110) and (220) planes, respectively (Figure S21). CsPbI_3_ films with different HTLs showed distinct behaviors after 6 h of aging (Figure S22). The bare CsPbI_3_ and CsPbI_3_/spiro‐OMeTAD films displayed a characteristic peak of the *δ* phase at 10.2°, indicating the transformation of the CsPbI_3_ perovskite structure to the yellow *δ* phase. This suggests that the dopants in spiro‐OMeTAD compromise the hydrophobic properties of the HTL, accelerating the phase transition of the CsPbI_3_ film. In contrast, the CsPbI_3_ films coated with dopant‐free HBT and HiBT HTLs retained the black phase, closely resembling the fresh film, indicating that HBT and HiBT effectively shield the metastable perovskite from moisture penetration. To assess the surface properties of the HTLs, we measured the contact angle of water droplets on the CsPbI_3_/HTL samples (Figure 6b). The contact angles of HBT and HiBT HTLs were approximately 79.8° and 92.8°, respectively, significantly higher than that of the CsPbI_3_ film (Figure S23).

We systematically evaluated the storage stability of PSCs under various conditions using different HTMs. For comparison, we also prepared CsPbI_3_ devices using doped and dopant‐free spiro‐OMeTAD, achieving PCEs of 20.28% and 9.43%, respectively (Figures S24 and S25). The unpackaged devices were stored in dark conditions within an argon‐filled glovebox. After 60 days, the devices with HBT and HiBT HTMs retained 94% and 100% of their initial performance, respectively, while the control device based on doped spiro‐OMeTAD maintained only 80% (Figure 6c). These results highlight that incorporating dopant‐free HTMs can reduce the mutual diffusion of ions, significantly improving the long‐term storage stability of PSCs.

To further investigate the thermal stability, the devices were tested at 85 °C under an argon atmosphere. As shown in Figure 6d, the PCE of the doped spiro‐OMeTAD PSC rapidly decreased to 30% of its initial value within 50 days. In contrast, the PSCs based on HBT and HiBT retained approximately 72% and 83% of their initial PCE after the same period, respectively. Additionally, the photothermal stability of the unpackaged devices was evaluated under white LED illumination at one sun intensity with MPP tracking at 85 °C. As depicted in Figure 6e, the HiBT‐based PSC maintained 80% of its initial efficiency after 1100 h, while the control PSC degraded rapidly within 200 h. This represents the highest photothermal stability reported for PSCs using dopant‐free small organic molecule HTMs. The superior thermal stability of the HiBT‐based PSC is attributed to both the higher *T*_g_ and passivating properties of the HiBT HTM. These results indicate that the HiBT HTM can stabilize the perovskite structure and inhibit ion migration, thus enhancing the thermal and photothermal stability of CsPbI_3_ PSCs.

## Conclusion

In this work, we successfully developed two isomeric D–A–D type small molecules using a symmetry‐breaking strategy and thoroughly investigated their performance as dopant‐free hole transport materials in inorganic CsPbI_3_ PSCs. Systematic studies reveal that the benzo[*d*][1,2,3]thiadiazole‐induced asymmetric structure effectively regulates the molecular dipole moment, enhances hole mobility, and lowers the HOMO energy level, which facilitates charge separation and contributes to a higher *V*_OC_ in PSCs. As a result, CsPbI_3_ devices with HiBT HTM achieved a champion PCE of 20.58%, marking the highest reported efficiency for all‐inorganic PSCs based on dopant‐free HTMs in the literature and notably surpassing the performance of those utilizing HBT HTMs. More importantly, the use of HiBT HTM, with its high hole mobility and *T*_g_, effectively mitigates the detrimental effects of dopants on the perovskite layer, preserving the high‐quality morphology of the HTL under photothermal conditions. Consequently, the dopant‐free HiBT HTM PSCs exhibited excellent long‐term stability, maintaining 83% of their initial PCE after approximately 50 days in darkness and 80% of their initial PCE after 1100 h under MPP tracking in an argon‐filled glovebox. This study not only offers a new framework for designing dopant‐free HTMs but also underscores the critical role of molecular engineering in optimizing both the efficiency and stability of n‐i‐p inorganic PSCs, thus paving the way for their large‐scale commercialization.

## Author Contributions

T.J., C.D., R.M., and G.L. proposed the research and designed the experiments. T.J. synthesized the HTMs. C.D., H.C., Q.Z., and R.M. fabricated the PSCs. T.P. conducted the simulated calculation. L.W. and X.G. measured and analyze the SCLC data. L.Y. and R.L. carried out the experiments of GIWAXS measurements. L.X., S.L. (Sha Liu), J.W., K.Y., and S.L. (Shengjian Liu) provided the experiment condition. C.D. and T.J. wrote the manuscript. All authors commented on the manuscript.

## Conflict of Interests

The authors declare no conflict of interest.

## Supporting information



Supporting Information

## Data Availability

The data that support the findings of this study are available from the corresponding author upon reasonable request.
