# Supplementary material for: Symmetry‐Breaking Strategy Yields Dopant‐Free Small Molecule Hole Transport Materials for Inorganic Perovskite Solar Cells with 20.58% Efficiency and Outstanding Stability
Source: Angew Chem Int Ed Engl. 2025 Apr 11;64(23):e202502478. doi: 10.1002/anie.202502478 (PMC12124450; doi:10.1002/anie.202502478)
Supplement: Supplementary file 1 — Supporting Information [file ANIE-64-e202502478-s001.docx]

Supporting Information

**Symmetry-Breaking Strategy Yields Dopant-Free Small Molecule Hole Transport Materials for Inorganic Perovskite Solar Cells with 20.58% Efficiency and Outstanding Stability**

*Huimin Cai,^a,†^ Qiliang Zhu,^b,†^ Tianchen Pan,^c,†^ Lunbi Wu,**^a^ Xin Gu,^a^ Chenghao Duan,^b,d,^* Liangbin Xiong,^a^ Jiaying Wu,^c^ Sha Liu,^e^ Liyang Yu,^f,g^ Ruipeng Li,^h^ Keyou Yan,^b^ Ruijie Ma,^i,^* Shengjian Liu,^j^ Tao Jia,^a,^* and Gang Li ^i,^**

*^a^*School of Optoelectronic Engineering, Guangdong Polytechnic Normal University, Guangzhou, 510665, P. R. China

*^b^*School of Environment and Energy, State Key Laboratory of Luminescent Materials and Devices, Guangdong Provincial Key Laboratory of Solid Wastes Pollution Control and Recycling, South China University of Technology, Guangzhou, 510000 P. R. China

*^c^*Advanced Materials Thrust, Function Hub, The Hong Kong University of Science and Technology (Guangzhou), Nansha 511400, Guangzhou, P. R. China

*^d^*Longzihu New Energy Laboratory, School of Energy Science and Technology, Henan University, Zhengzhou, 450000, P. R. China

*^e^*Dongguan Key Laboratory of Interdisciplinary Science for Advanced Materials and Large-Scale Scientific Facilities, School of Physical Sciences, Great Bay University, Dongguan, Guangdong, 523000, P. R. China

*^f^*Research Institute of Frontier Science, Southwest Jiaotong University, Chengdu 610031, P. R. China

*^g^*School of Chemical Engineering, Sichuan University, Chengdu 610065, P. R. China

*^h^*National Synchrotron Light Source (NSLS II), Brookhaven National Laboratory, Upton, NY, 11973

*^i^*Department of Electrical and Electronic Engineering, Research Institute for Smart Energy (RISE), Photonic Research Institute (PRI), Guangdong-Hong Kong-Macao Joint Laboratory for Photonic-Thermal-Electrical Energy Materials and Devices, The Hong Kong Polytechnic University, Hong Kong, China

*^j^*School of Chemistry, Guangzhou Key Laboratory of Materials for Energy Conversion and Storage, Key Laboratory of Electronic Chemicals for Integrated Circuit Packaging, South China Normal University (SCNU), Guangzhou 510006, P. R. China

^†^These authors contributed equally.

***Corresponding author

E-mail: chduan2025@henu.edu.cn (C. Duan); [ruijie.ma@polyu.edu.hk](mailto:ruijie.ma@polyu.edu.hk) (R. Ma); [tjia@gpnu.edu.c](mailto:ruijie.ma@polyu.edu.hk)n (T. Jia); [gang.w.li@polyu.edu.hk](mailto:gang.w.li@polyu.edu.hk) (G. Li)

**Experimental Section**

**Materials**

Methoxy-N-(4-methoxyphenyl)-N-(4-(4,4,5,5-tetramethyl-1,3,2-dioxaborolan-2-yl)phenyl)aniline (TPA-B) was purchased from Jiangsu Aikon. 4,7-Dibromobenzo[c][1,2,5]thiadiazole (BrBT) was purchased from Bide Pharmatech Ltd. 4,7-Dibromobenzo[d][1,2,3]thiadiazole (BriBT) was synthesized as our reported literature.^[1]^ Unless otherwise noted, commercial reagents were purchased from GUANGZHOU Chemical and used without further purification.

**Materials characterization**

The ^1^H NMR and ^13^C NMR spectra were measured on a Bruker AVANCE NEO (600 MHz) spectrometer with tetramethylsilane (TMS) as the internal reference at room temperature. Mass spectra were measured on Bruker ultrafleXtreme or Bruker tims-TOF instrument.

**General Experimental Details**

**Scheme S1**. The synthesis routes of HiBT and HBT.

*Synthesis of 4,4'-(benzo[d][1,2,3]thiadiazole-4,7-diyl)bis(N,N-bis(4-methoxyphenyl)aniline) (****HiBT****)*

Compound TPA-B (1.034 g, 2.4 mmol), BriBT (294 mg, 1 mmol), Pd(PPh_3_)_4_ (57.8 mg, 0.05 mmoL), toluene (15 mL) and K_2_CO_3_ (aq. 2M, 2 mL) were added into a pressure-proof pipe under argon atmosphere. The reaction mixture was stirred at reflux overnight. After cooling to room temperature, The reaction mixture was evaporated under reduced pressure to remove toluene solvent. The residue was purified by column chromatography with dichloromethane (DCM)/ethyl acetate (EA) (*v*:*v*, 2/1~1/1) as eluent to afford compound **HiBT** as an yellow solid (683.2 mg, yield 92%). ^1^H NMR (600 MHz, CDCl_3_) δ 7.78 (d, *J* = 76.3 Hz, 4H), 7.49 (d, *J* = 7.8 Hz, 2H), 7.22 – 6.82 (m, 18H), 3.82 (s, 12H).^13^C NMR (151 MHz, CDCl_3_) δ 156.93, 156.36, 141.15, 131.80, 127.73, 127.31, 119.74, 114.85, 55.50. MALDI-TOF m/z calcd. for C_46_H_38_N_4_O_4_S, 742.894; found, 742.261.


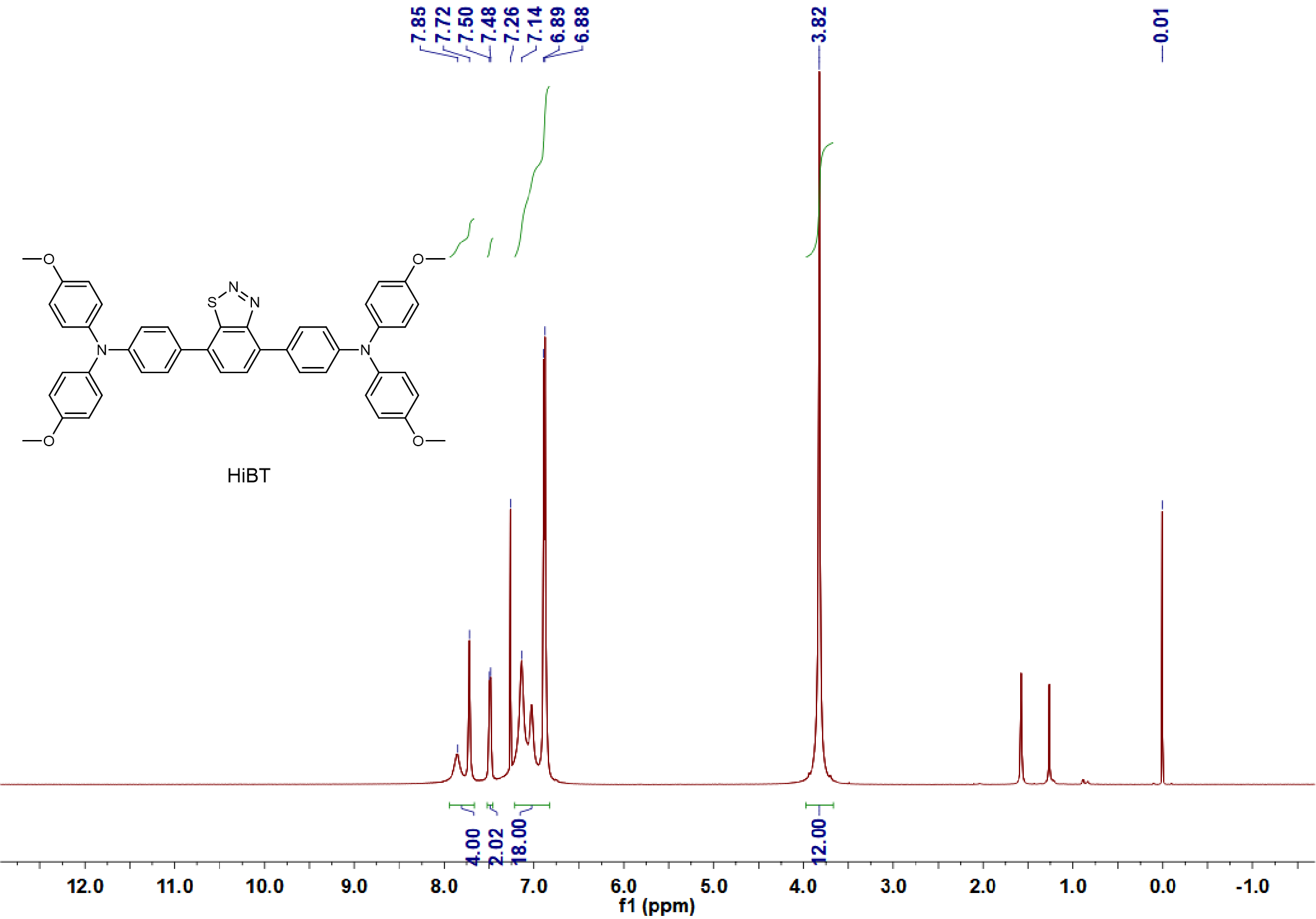


**Figure S1**. The ^1^H NMR spectrum of HiBT.


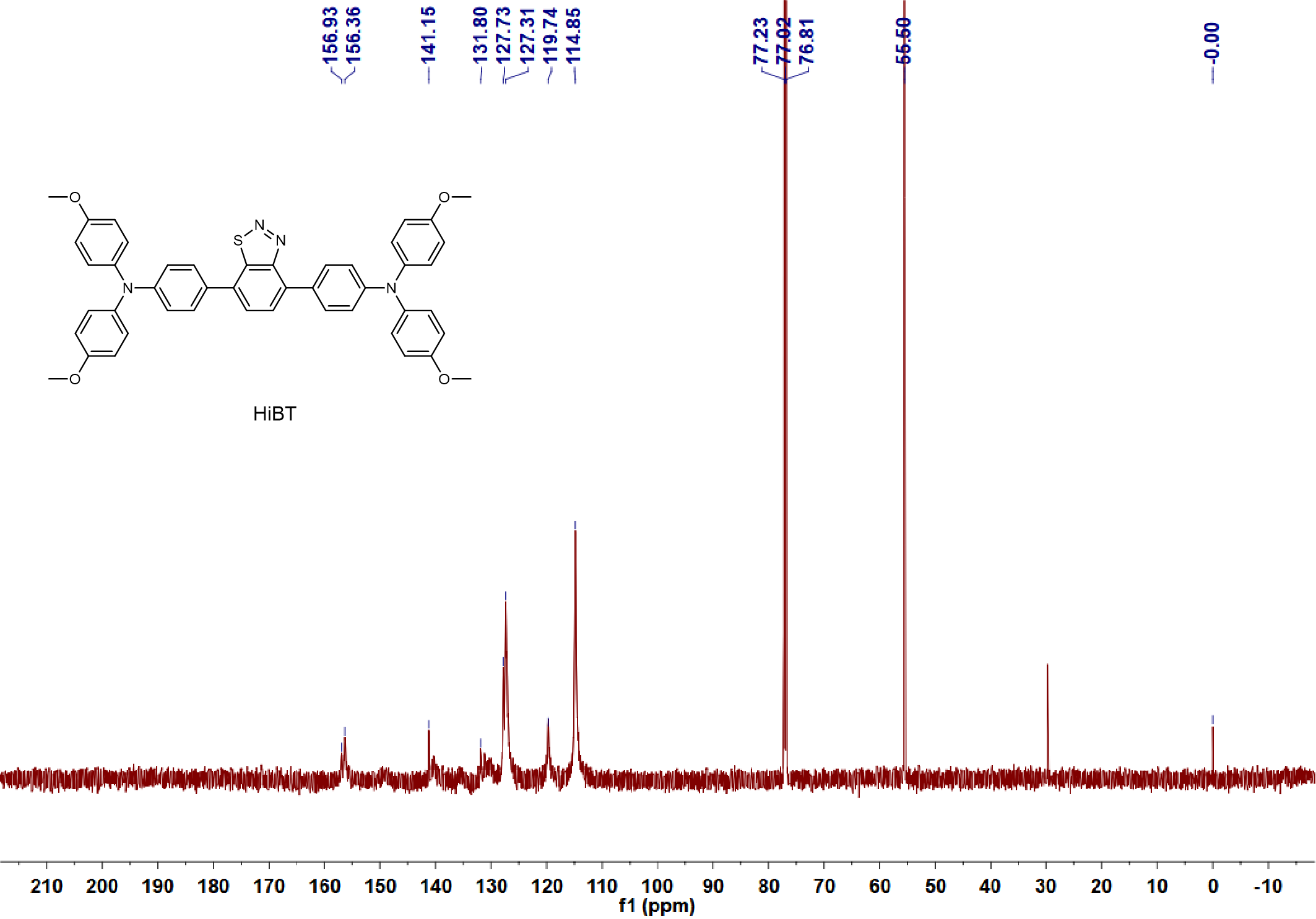


**Figure S2**. The ^13^C NMR spectrum of HiBT.

**Figure S3**. Mass spectrum of HiBT.

*Synthesis of 4,4'-(benzo[c][1,2,5]thiadiazole-4,7-diyl)bis(N,N-bis(4-methoxyphenyl)aniline) (****HBT****)*

The synthesis method of HBT is similar to that of HiBT, requiring only the substitution of BriBT with BrBT during the preparation process, yielding HBT as a purple solid (653.0 mg, yield 90%). ^1^H NMR (600 MHz, CDCl_3_) δ 7.82 (d, *J* = 8.5 Hz, 4H), 7.69 (s, 2H), 7.15 – 7.05 (m, 12H), 6.87 (d, *J* = 8.8 Hz, 8H). ^13^C NMR (151 MHz, CDCl_3_) δ 156.12, 154.23, 148.76, 140.59, 132.03, 129.68, 129.13, 127.09, 127.00, 119.83, 114.74, 55.50. MALDI-TOF m/z calcd. for C_46_H_38_N_4_O_4_S, 742.894; found, 742.260


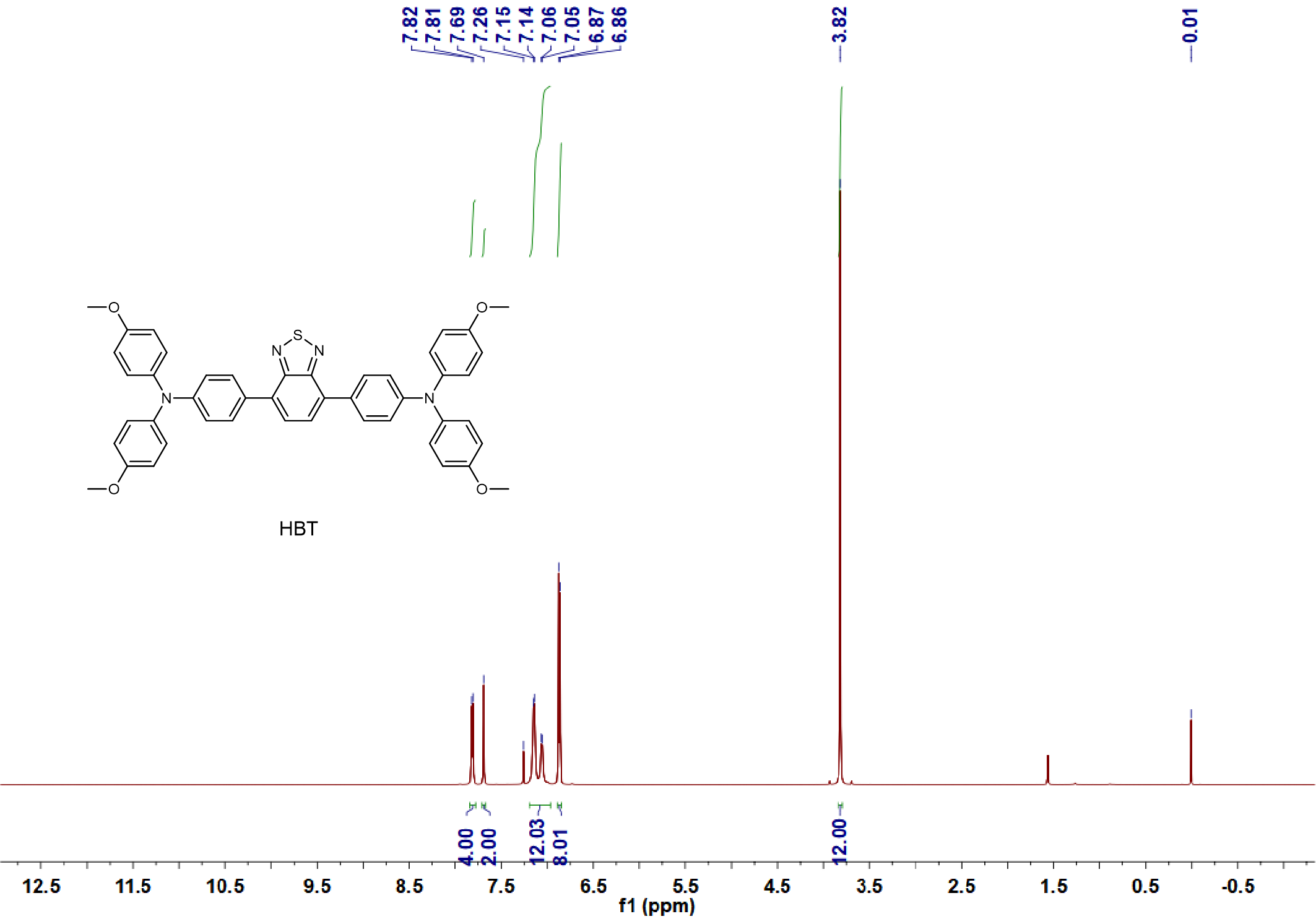


**Figure S4**. The ^1^H NMR spectrum of HBT.


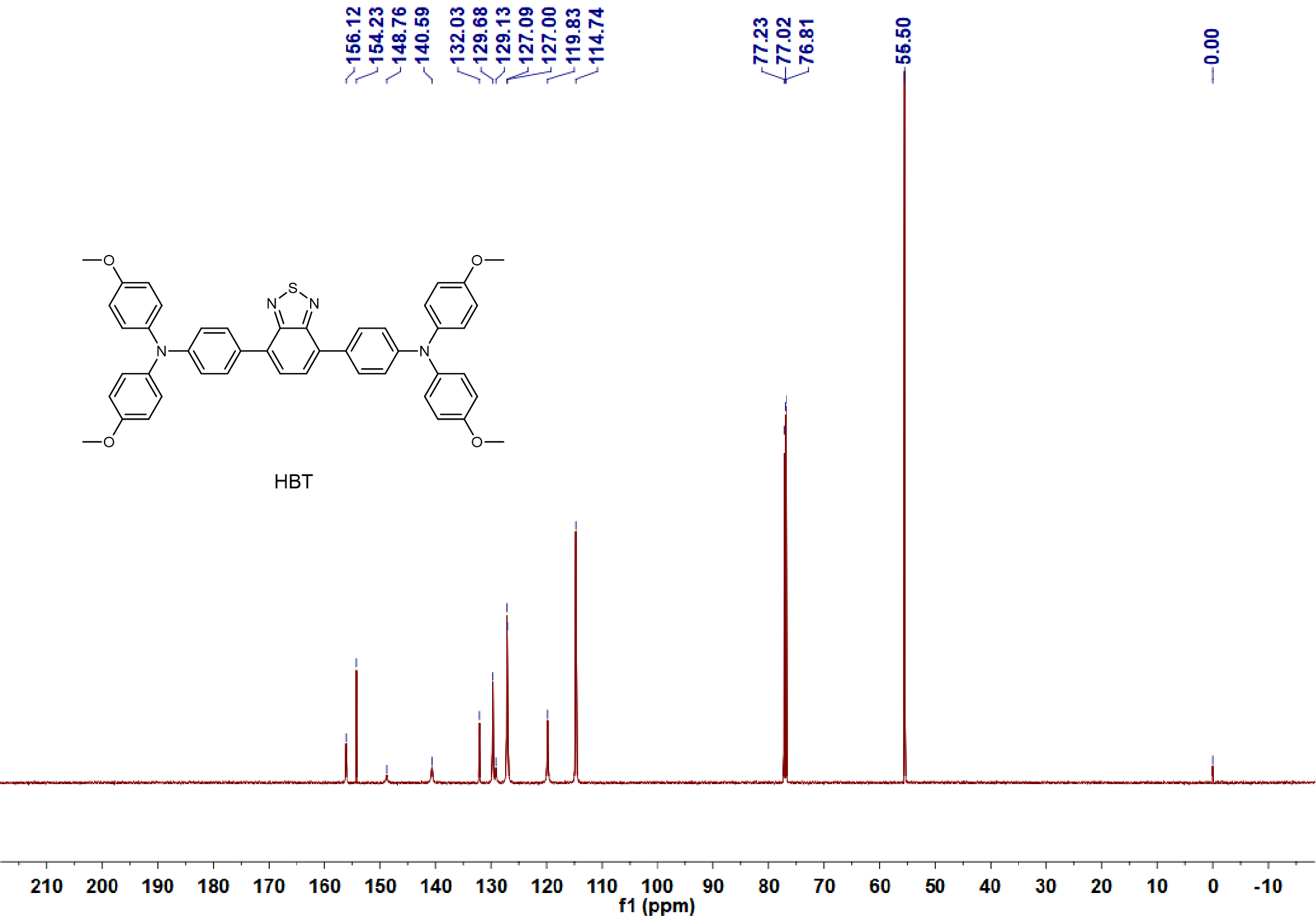


**Figure S5**. The ^13^C NMR spectrum of HBT.

**Figure S6**. Mass spectrum of HBT.

**Device Fabrication**

FTO glass substrate (Advanced Election Technology Co., Ltd) was sequentially cleaned using ultrasonic cleaning with detergent, deionized water, acetone, ethanol, and isopropanol for 15 minutes. Then, dry the FTO with nitrogen and treat it with ultraviolet ozone for 15 minutes. A 200 mL volume of deionized water was placed into a glass dish and frozen, after which 4.5 mL of TiCl_4_ solution was slowly added to the ice water. Upon complete melting, the solution was poured into a glass dish containing the FTO substrate and heated in an oven at 80 °C for 1 h. The substrate was subsequently rinsed with deionized water and ethanol, followed by annealing at 200 °C for 5 min. Then, these substrates were transferred to an N_2_ glovebox for perovskite deposition. The perovskite layer was fabricated using a one-step spin-coating method. A precursor solution containing 0.7 M PbI_2_, 0.7 M DMAI, and 0.7 M CsI dissolved in 1 mL of dimethylformamide (DMF) was stirred at 60 °C for 8 h. The solution was then spin-coated onto the TiO_2_ layer at 3,500 rpm for 35 s, followed by annealing at 200 °C for 5 min. Varying concentrations of HBT and HiBT in CB solutions were deposited by spin-coating at 4000 rpm for 30 s. Finally, an 8 nm MoO_3_ blocking layer and a 100 nm metal electrode were deposited via vacuum thermal evaporation.

**Device Characterization**

Current density–voltage (*J*–*V*) characteristics of the PSCs were measured using a Keithley 2400 source measurement unit under simulated AM 1.5G illumination (100 mW/cm²). The light intensity was calibrated using a standard single-crystal silicon solar cell (SS-F5-3A solar simulator, Enli Technology Co., Ltd.). EQE measurements were conducted on a Zahner system equipped with a TLS03 light source (300 Hz, 100 counts) in mixed mode. Collect Photoluminescence (PL) spectra using a monochromator, separate the peak PL wavelength, and time-correlated single-photon counting (TCSPC) was conducted using a Becker and Hickl system. Time-resolved photoluminescence (TRPL) measurements were conducted on packaging films with a glass/perovskite/HTM structure. Thermogravimetric analysis (TGA) was carried out using a TA Instruments thermogravimetric analyzer at a heating rate of 15 °C/min under a nitrogen atmosphere. Differential scanning calorimetry (DSC) was performed on a DSC201F2 instrument (NETZSCH) under nitrogen protection at a heating rate of 10 °C/min.

**Calculation Methods**

**Electrostatic potential (ESP)**

The molecular electrostatic potential (ESP), V(r), has been widely used for the prediction of nucleophilic and electrophilic sites, as well as molecular recognition mode for a long time, the theoretical basis is that molecules always tend to approach each other in a complementary manner of ESP. These analyses of ESP are commonly performed on molecular van der Waals (vdW) surfaces. Although the definition of such a surface is arbitrary, most people are prone to take the 0.001 (a.u.) isosurface of electron density as vdW surface, since this definition reflects specific electron structure features of a molecule, such as lone pairs and π electrons, this is also what the definition used in our analyses.


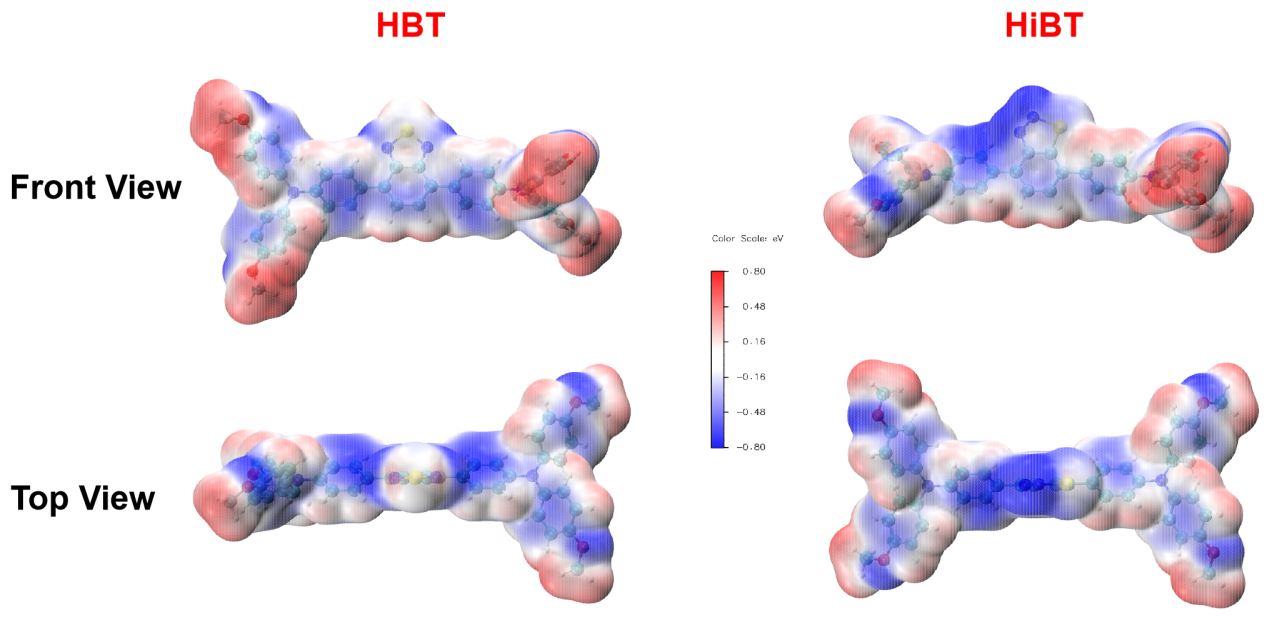


**Figure S7**. The front and top views of electrostatic potential distributions of HBT and HiBT.

**DFT calculation**

Gaussian 16 (Revision C.02) code^[2]^ was used for density functional theory (DFT) calculations, including structure optimization and single-point energy calculations. For structure optimization, the B3LYP-D3(BJ)/defTZVP^[3-5]^ level of theory was applied. For single-point calculations of the complexes, the B3LYP-D3(BJ)/Def2TZVP level was employed to balance accuracy with reduced computational cost and time. The Wavefunction software Multiwfn^[6]^ and VMD^[7]^ were used to analyze the electrostatic potential (ESP) and the electron-hole (e-h) distribution.


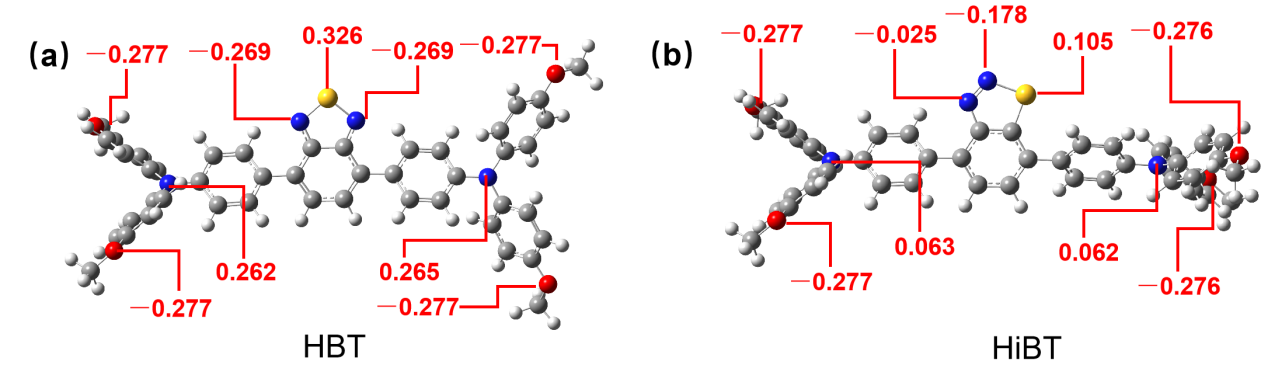


**Figure S8**. The main atomic charges of of HBT and HiBT molecules.


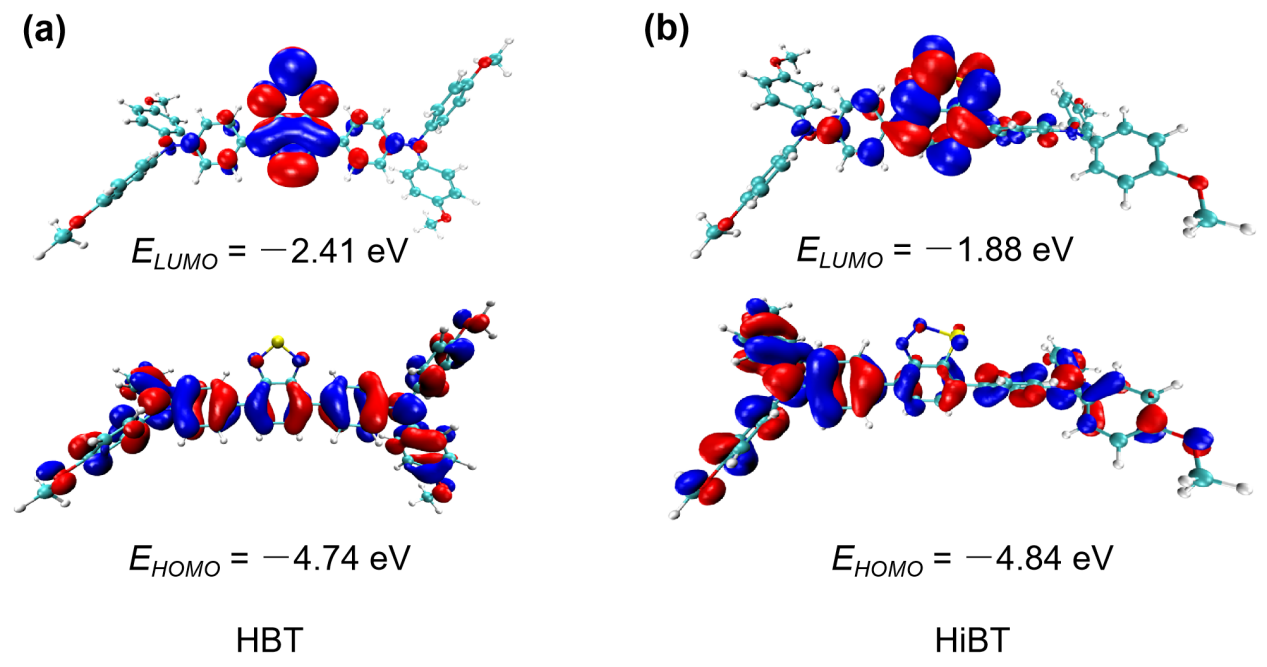


**Figure S9**. The HOMO and LUMO distributions and energy levels of (a) HBT and (b) HiBT.

**Hole-electron distribution**

Excited state wavefunction ($\Psi^{excited}$) of TD-DFT type can be represented as follows:

$$\Psi^{excited}=\underset{i\to a}{\sum}w_{i}^{a}\Phi_{i}^{a}+\underset{i\leftarrow a}{\sum}w_{i}^{'a}\Phi_{i}^{a}$$

where *i* and *a* respectively run over all occupied and all virtual MOs. $\Phi_{i}^{a}$ is the configuration state wavefunction corresponding to moving an electron from originally occupied *i* MO to virtual *a* MO. *w* and *w'* correspond to configuration coefficients of excitation and de-excitation, respectively.

**HTM/Perovskite Defect Passivation Simulation**

Simulations were performed using VASP 6.4.1, employing the generalized gradient approximation (GGA) with the Perdew-Burke-Ernzerhof (PBE) formulation. ^[8]^ The projected augmented wave (PAW) potentials ^[9]^ was used to describe the ionic cores, with a plane-wave basis set and a kinetic energy cutoff of 450 eV. Partial occupancies of the Kohn-Sham orbitals were treated using the Gaussian smearing method with a width of 0.05 eV. The electronic energy was considered self-consistent when the energy change was below 10^-6^ eV. A vacuum level of 25 Å was set along the z-axis, and Brillouin zone integration was performed using Gamma point 1×1×1 k-point sampling. Input files were generated using the VASPkit tool. ^[10]^


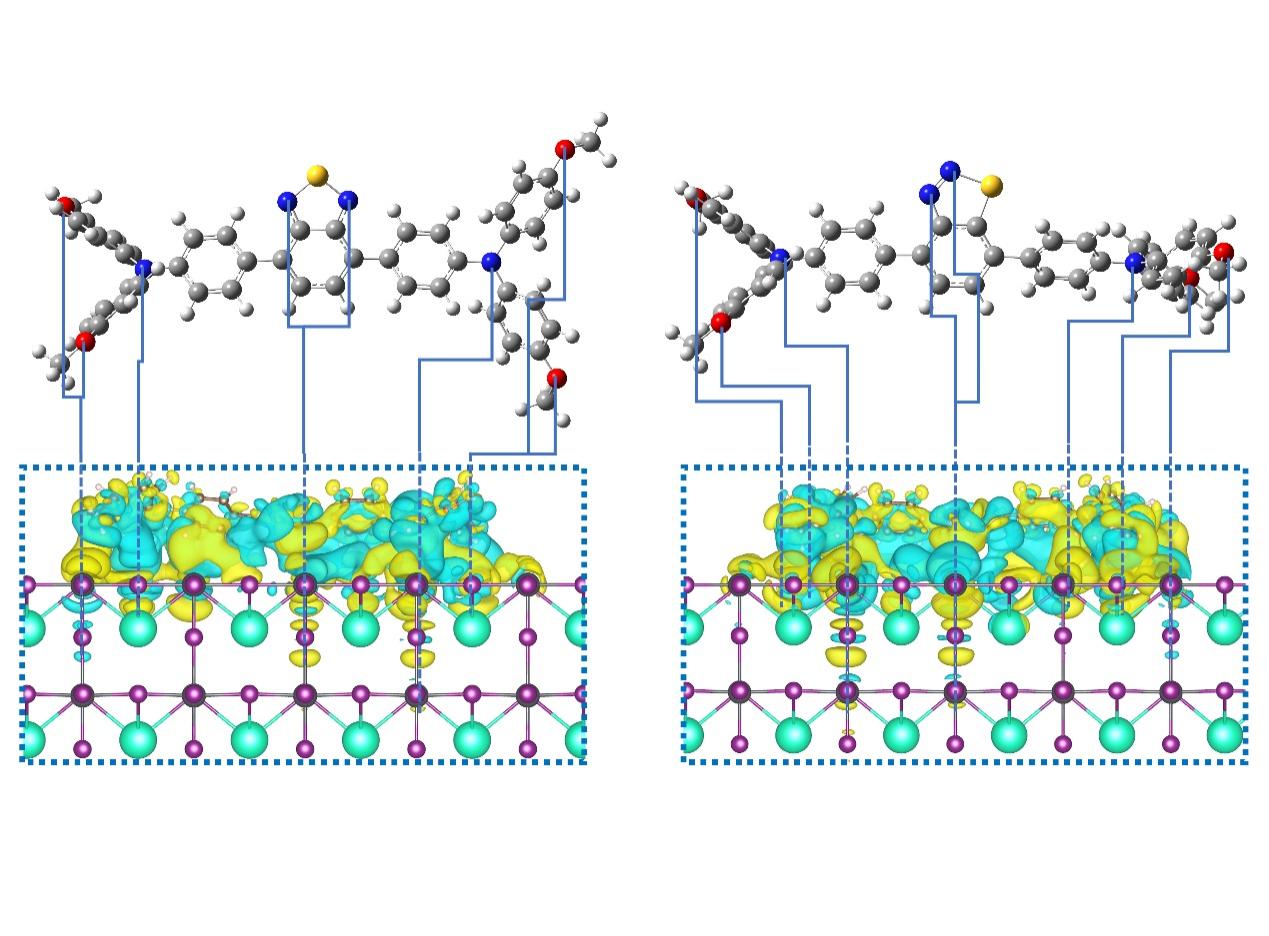


**Figure S10**. Total electron density differences of (a) HBT/perovskite and (b) HiBT/perovskite.

**Thermal Property Analyses**

Thermogravimetric (TG) measurements were conducted using a NETZSCH TG209F3 apparatus, with a heating rate of 10 °C/min under a nitrogen atmosphere. Differential scanning calorimetry (DSC) analysis was conducted using a NETZSCH DSC200F3 apparatus, with a heating and cooling rate of 10 °C/min under a nitrogen atmosphere.


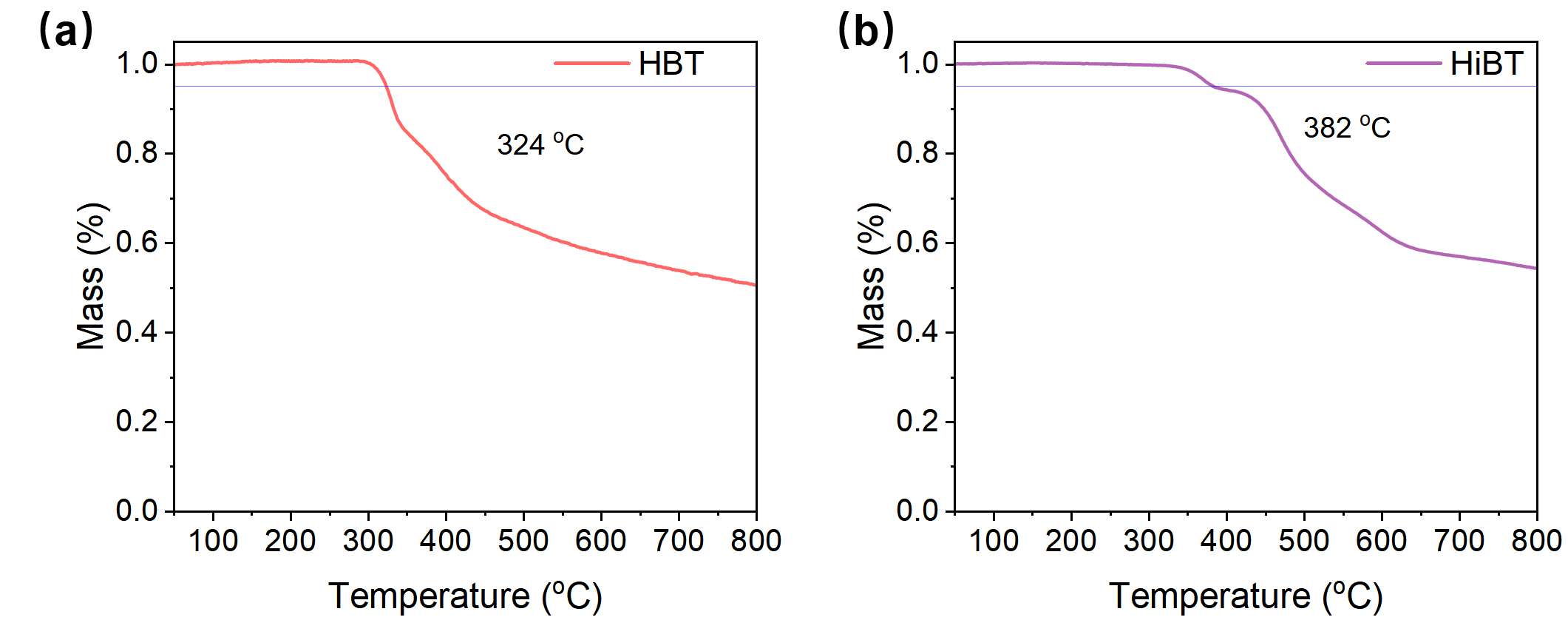


**Figure S11.** Thermogravimetric analysis (TGA) curves for HBT, and HiBT.


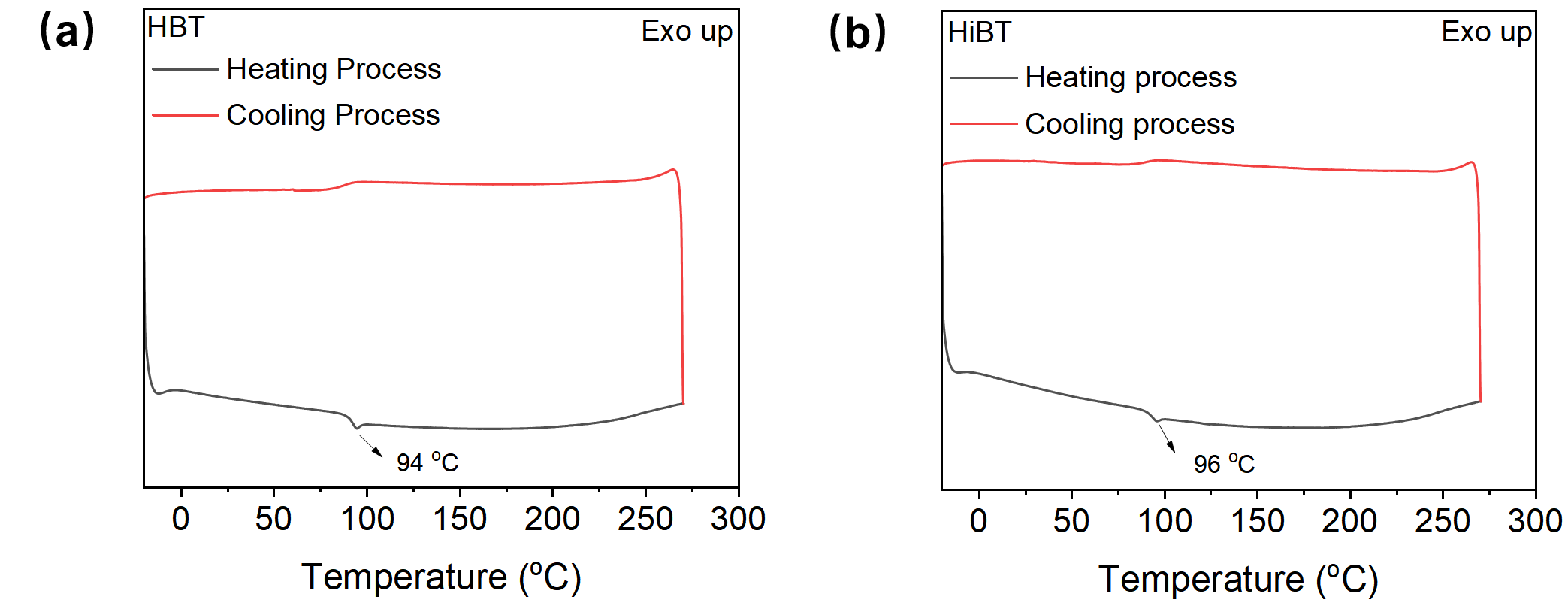


**Figure S12.** DSC curves of HBT and HiBT.

**SCLC Measurements**

The charge carrier mobilities of HTL films are estimated from space-charge-limited current (SCLC) method. The hole-only devices were fabricated with the architectures of ITO/PEDOT:PSS/HTLs/MoO_3_/Ag. Hole-only devices were recorded with a Keithley 236 source meter under dark. The hole mobilities were determined by fitting the dark current to the model of single-carrier SCLC, which is described by the equation,

$$J=\frac{9}{8}\varepsilon_{0}\varepsilon_{\gamma}\mu\frac{V^{2}}{d^{3}}$$

where *J* is the current density, *μ* is the zero-field mobility, *ε*_0_ is the permittivity of free space, *ε*_r_ is the relative permittivity of the material, *d* is the thickness of the active layers, and *V* is the effective voltage. The effective voltage was obtained by subtracting the built-in voltage (*V*_bi_) and the voltage drop (*V*_s_) from the series resistance of the whole device except for the active layers from the applied voltage (*V*_appl_), *V* = *V*_appl_ − *V*_bi_ − *V*_s_. (*V*_bi_ = 0 and *V*_s_ =10×*I*, where the value 10 is the resistance of MoO_3_ and *I* is the current of the devices in this work). The hole mobilities can be calculated from the slope of the *J*^1/2^*-V* curves.

**
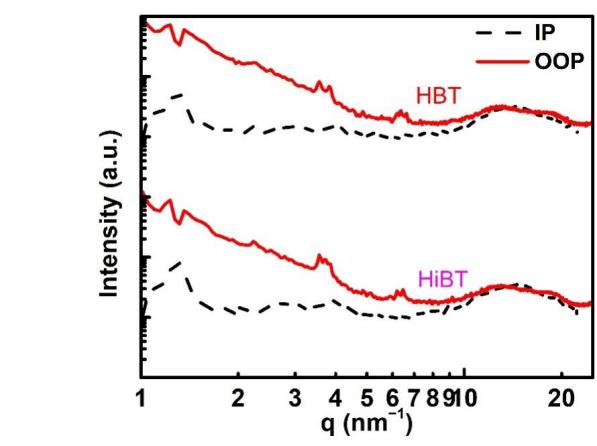
**

**Figure S13.** The corresponding GIWAXS intensity profiles of HBT and HiBT films.

**
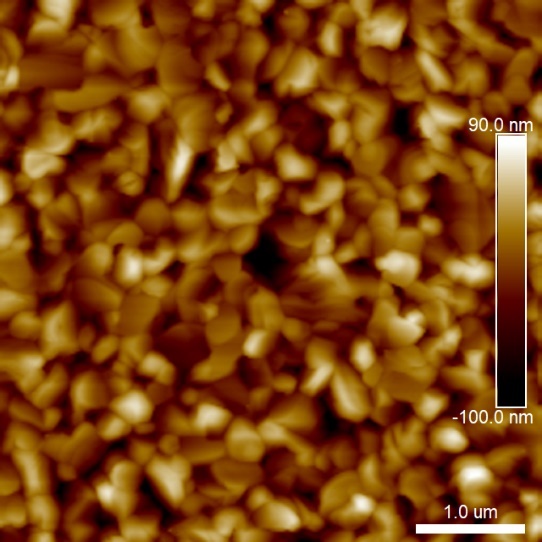
**

**Figure S14.** The AFM image of CsPbI_3_ perovskite film.

**
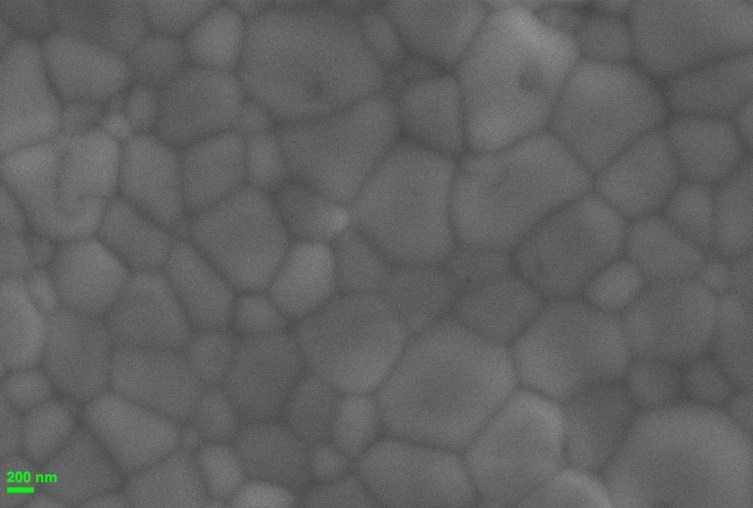
**

**Figure S15.** The SEM image of CsPbI_3_ perovskite film.

**
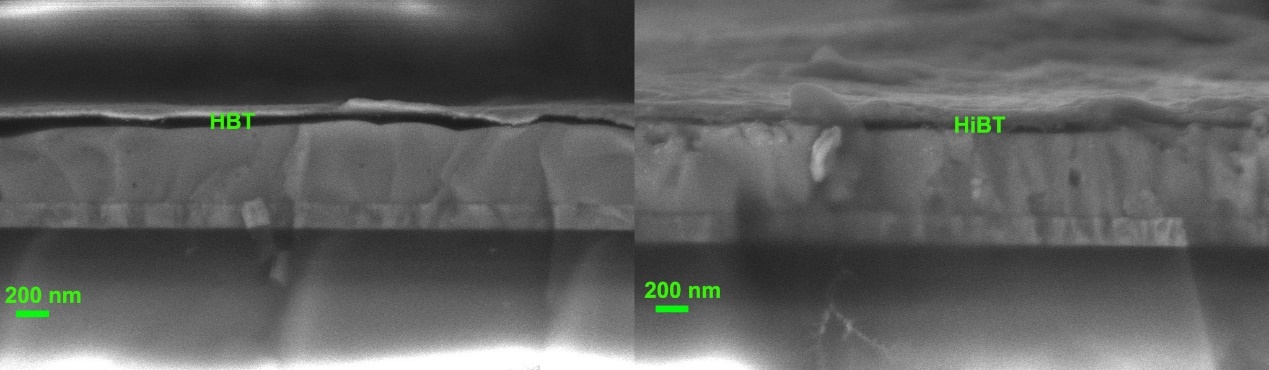
**

**Figure S16.** The cross-section SEM images of the devices based on HBT and HiBT HTMs.

**
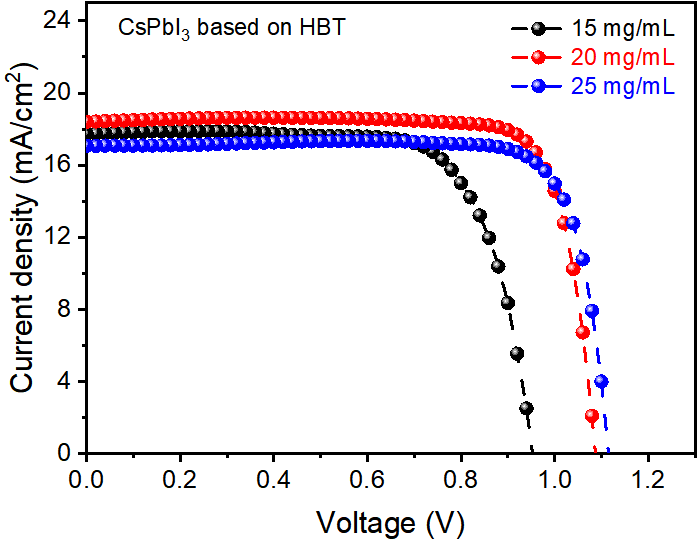
**

**Figure S17.** *J*-*V* characteristic curves of the n-i-p CsPbI_3_ solar cells based on BT with different concentrations under AM 1.5G 100 mW/cm^2^ simulated solar light.

**
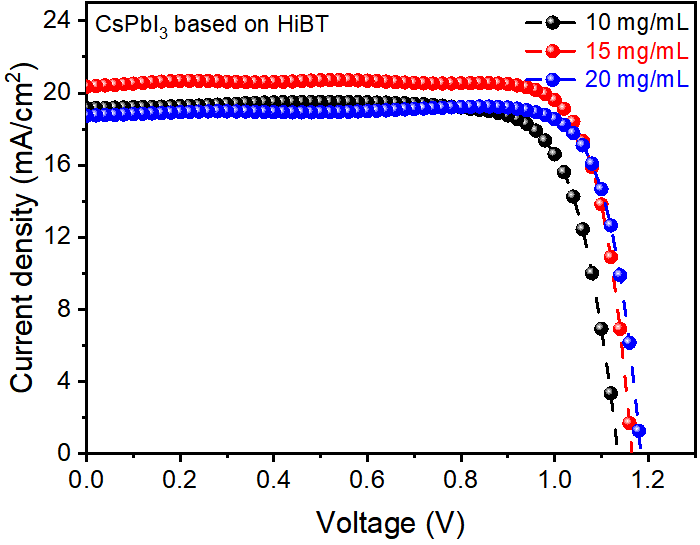
**

**Figure S18.** *J*-*V* characteristic curves of the n-i-p CsPbI_3_ solar cells based on iBT with different concentrations under AM 1.5G 100 mW/cm^2^ simulated solar light.


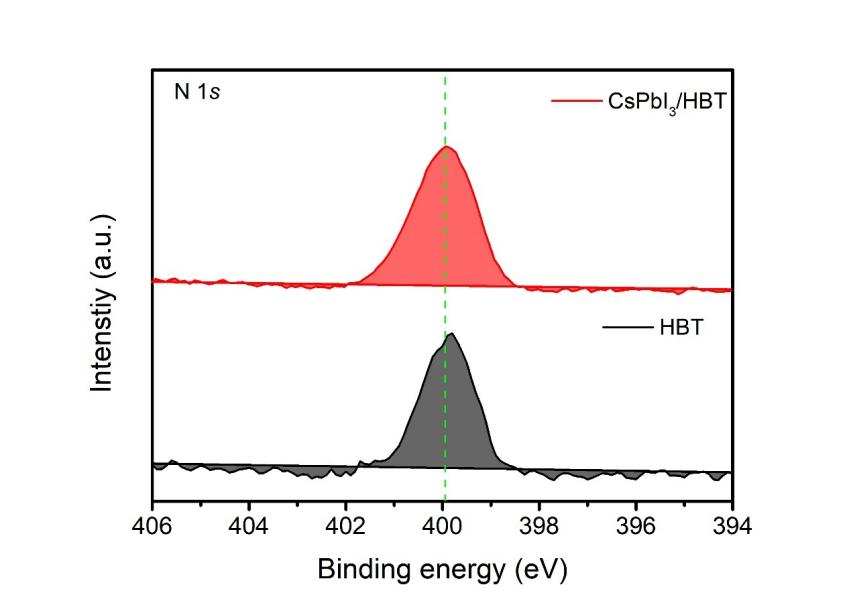


**Figure S19.** XPS N 1*s* spectra of HBT and CsPbI_3_/HBT films.


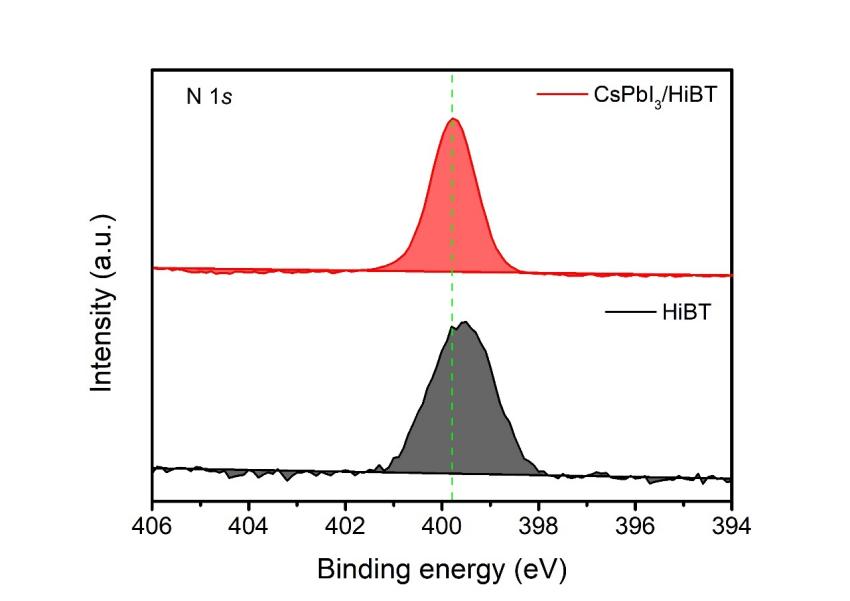


**Figure S20.** XPS N 1*s* spectra of HiBT and CsPbI_3_/HiBT films.


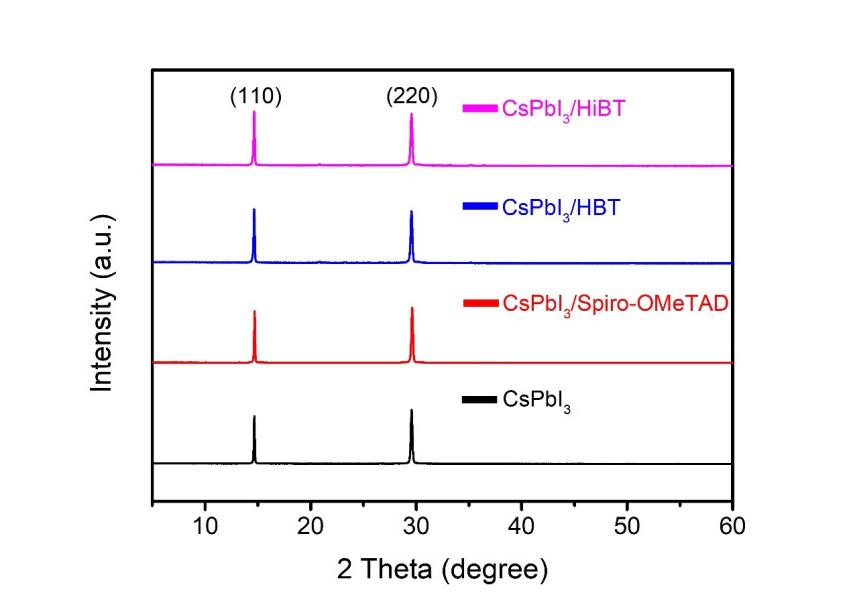


**Figure S21.** XRD pattern of the CsPbI_3_ films with and without different HTLs before exposure to an RH of 50 ± 5%.


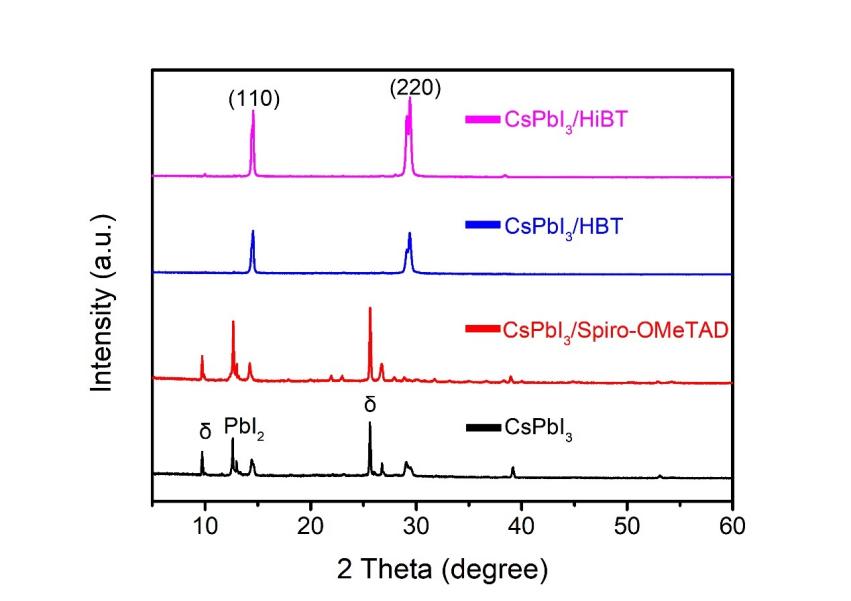


**Figure S22.** XRD pattern of the CsPbI_3_ films with and without different HTLs after exposure to an RH of 50 ± 5% for 6 h.


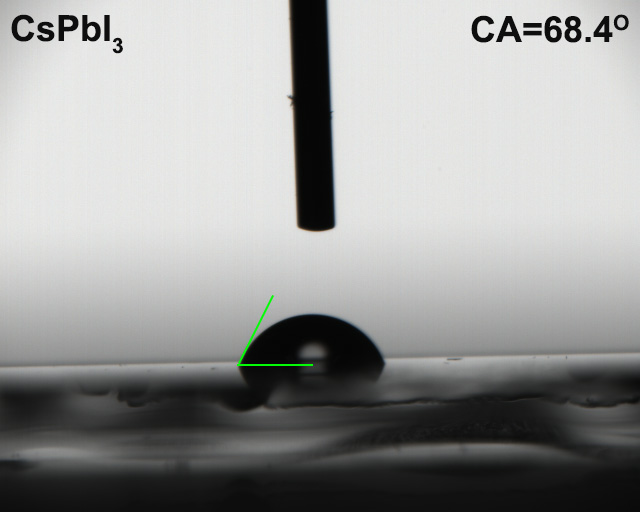


**Figure S23.** The Contact angles of CsPbI_3_ perovskite film.


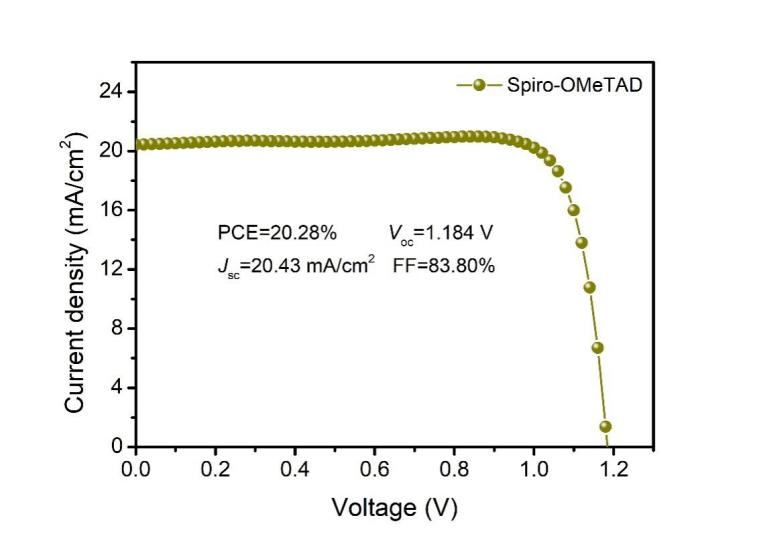


**Figure S24.** *J*-*V* characteristic curves of the n-i-p CsPbI_3_ solar cells based on doped Spiro-OMeTAD under AM 1.5G 100 mW/cm^2^ simulated solar light.


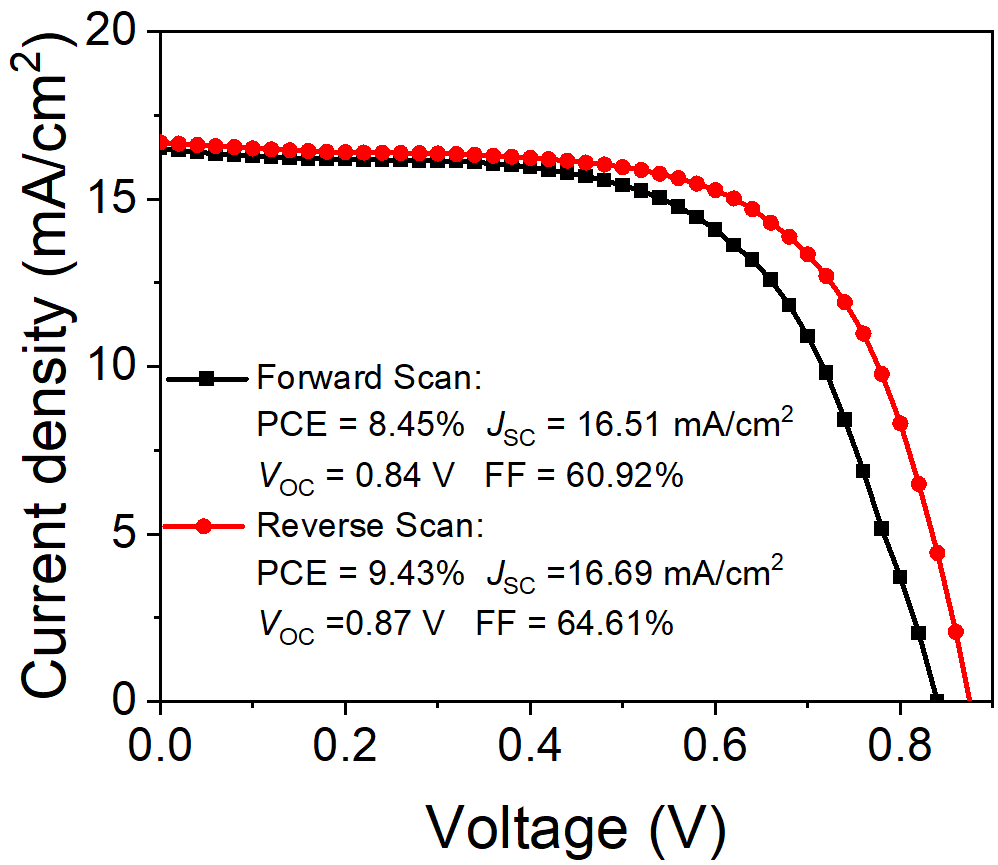


**Figure S25**. *J-V* curves of the CsPbI_3_ devices based on dopant-free Spiro-OMeTAD as HTL.

**Estimated costs of HiBT and HBT**

The synthetic cost of HiBT and HBT are estimated to be 74.51 CNY/g (10.16 USD/g) and 77.15 CNY/g (10.52 USD/g). For comparison, the price of Spiro-OMeTAD collected from https://www.chemicalbook.com//(accessed: Jan. 2025) are 118.19 (aladdin chemical) ~ 611.79 (Sigma-addrich chemical) USD/g). CNY: Chinese Yuan; USD: US Dollar.

**Table S1** Estimated synthetic cost of HiBT and HBT with 1g scale.

| HTMs | Chemical | Chemical brand | Weight (g) | Volume (L) | Price  (CNY for 1g or 1 L) | Cost (CNY) |
| --- | --- | --- | --- | --- | --- | --- |
| HiBT | 4-Methoxy-N-(4-methoxyphenyl)-N-(4-(4,4,5,5-tetramethyl-1,3,2-dioxaborolan-2-yl)phenyl)aniline (TPA-B) | Jiangsu Aikon | 1.51 | － | 27.22 | 41.10 |
|  | 4,7-Dibromobenzo[d][1,2,3]thiadiazole (BriBT) | － | 0.43 | － | 0.631 | 0.27 |
|  | Pd(PPh_3_)_4_ | Bidepharm | 0.085 | － | 41.6 | 3.54 |
|  | Toluene | GUANGZHOU | － | 0.05 | 76 | 3.80 |
|  | Petroleum ether | GUANGZHOU | － | 0.5 | 20 | 10 |
|  | CH_2_Cl_2_ | GUANGZHOU | － | 0.2 | 30 | 6 |
|  | CH_3_COOC_2_H_5_ | GUANGZHOU | － | 0.1 | 24 | 2.4 |
|  | CH_3_OH | GUANGZHOU | － | 0.1 | 24 | 2.4 |
|  | Silica gel | Qingdao Haiyang | 50 | － | 0.1 | 5.0 |
| Total cost (CNY) | － | － | － |  | － | 74.51 |
| HBT | 4-Methoxy-N-(4-methoxyphenyl)-N-(4-(4,4,5,5-tetramethyl-1,3,2-dioxaborolan-2-yl)phenyl)aniline (TPA-B) | Jiangsu Aikon | 1.55 | － | 27.22 | 42.2 |
|  | 4,7-Dibromobenzo[c][1,2,5]thiadiazole (BrBT) | Bidepharm | 0.440 |  | 4.12 | 1.81 |
|  | Pd(PPh_3_)_4_ | Bidepharm | 0.085 |  | 41.6 | 3.54 |
|  | Toluene | GUANGZHOU | － | 0.05 | 76 | 3.80 |
|  | Petroleum ether | GUANGZHOU | － | 0.5 | 20 | 10 |
|  | CH_2_Cl_2_ | GUANGZHOU | － | 0.2 | 30 | 6 |
|  | CH_3_COOC_2_H_5_ | GUANGZHOU | － | 0.1 | 24 | 2.4 |
|  | CH_3_OH | GUANGZHOU | － | 0.1 | 24 | 2.4 |
|  | Silica gel | Haiyang (Qingdao) | 50 | － | 0.1 | 5.0 |
| Total cost (CNY) | － | － | － |  | － | 77.15 |

**Table S2.** Device parameters of conventional n-i-p CsPbI_3_ solar cells based on HBT with different concentrations.

| Samples | *V*_oc_(V) | *J*_sc_(mA/cm^2^) | FF(%) | PCE(%) |
| --- | --- | --- | --- | --- |
| 15 mg/mL | 0.95 | 17.72 | 73.42 | 12.39 |
| 20 mg/mL | 1.09 | 18.39 | 81.39 | 16.28 |
| 25 mg/mL | 1.11 | 17.06 | 81.43 | 15.49 |

**Table S3.** Device parameters of conventional n-i-p CsPbI_3_ solar cells based on HiBT with different concentrations.

| Samples | *V*_oc_(V) | *J*_sc_(mA/cm^2^) | FF(%) | PCE(%) |
| --- | --- | --- | --- | --- |
| 10 mg/mL | 1.14 | 18.42 | 81.52 | 17.10 |
| 15 mg/mL | 1.16 | 20.35 | 82.72 | 19.61 |
| 20 mg/mL | 1.18 | 18.75 | 83.80 | 18.60 |

**Table S4.** Performance parameters of inorganic perovskite solar cells based on doped-free HTMs

| HTM type | HTM | Device structure | V_oc_ (V) | J_sc_ (mA/cm^2^) | FF (%) | PCE (%) | Ref. |
| --- | --- | --- | --- | --- | --- | --- | --- |
| Polymer | PM6 | ITO/ZTiO_2_/CsPbI_3_:/PM6/MoO_3_/Ag | 1.08 | 20.47 | 82.8 | 18.27 | 11 |
|  | PTB7 | FTO/TiO_2_/CsPbI_3_ QDs/PTB7/MoO_3_/Ag | 1.27 | 12.39 | 80 | 12.55 | 12 |
|  | PTQ10 | ITO/ SnO_2_/CsPbI_2_Br/PTQ10/MoO_3_/Ag | 1.40 | 15.24 | 83.2 | 17.8 | 13 |
|  | poly(DTSTPD-r-BThTPD) | FTO/TiO_2_/CsPbI_2_Br/poly(DTSTPD-r-BThTPD)/Au | 1.15 | 15.66 | 81 | 14.58 | 14 |
|  | P3HT | FTO/TiO_2_/CsPbI_2_Br/P3HT/Au | 1.303 | 15.90 | 75.76 | 15.69 | 15 |
|  | PCDA1 | FTO/SnO_2_/CsPbI_2_Br/PCDA1/Au | 1.07 | 14.55 | 69 | 11.01 | 16 |
|  | PE65 | ITO/SnO_2_/CsPbI_2_Br/PE65/MoO_3_/Ag | 1.44 | 14.54 | 84.08 | 17.60 | 17 |
|  | PFBTI | ITO/SnO_2_/CsPbI_3-x_Br_x_/PFBTI/MoO_3_/Ag | 1.3 | 16.3 | 82.1 | 17.4 | 18 |
|  | PDCBT | ITO/SnO_2_/PN4N/CsPbI_2_Br/PDCBT/MoO_3_/Ag | 1.30 | 15.3 | 81.5 | 16.2 | 19 |
|  | PM6 | ITO/SnO_2_/ZnO/CsPbI_2_Br/PM6/MoO_3_/Ag | 1.241 | 15.68 | 82.54 | 16.06 | 20 |
|  | PBDB-T | ITO/TiO_2_/SnO_2_/CsPbI_2_Br/PBDB-T/MoO_3_/Ag | 1.24 | 16.16 | 82.05 | 16.40 | 21 |
|  | PSQ2 | ITO/ZnO/SnO_2_/CsPbI_2_Br/PSQ2/MoO_3_/Ag | 1.27 | 15.4 | 79.0 | 15.5 | 22 |
|  | PBDB-T-Si | ITO/ZnO/CsPbI_2_Br/PBDB-T-Si/MoO_3_/Ag | 1.20 | 15.47 | 84.03 | 15.60 | 23 |
|  | PCPDTBT | ITO/SnO_2_/ZnO/CsPbI_2_Br/PCPDTBT/ MoO_3_/Ag | 1.275 | 15.49 | 83.52 | 16.49 | 24 |
| Small-molecule | CI-TTIN-2F | ITO/TiO_2_/CsPbI_3_/CI-TTIN-2F/Au | 1.10 | 18.82 | 77.50 | 15.9 | 25 |
|  | BD | ITO/TiO_2_/CsPbI_3_/BD/MoO_x_/Ag | 1.12 | 20.87 | 81.97 | 19.19 | 26 |
|  | L2 | ITO/SnO_2_/CsPbI_2_Br/L_2_/MoO_3_/Ag | 1.21 | 13.73 | 75.02 | 12.41 | 27 |
|  | YN3 | FTO/TiO_2_/CsPbI_2_Br/YN3/Au | 1.12 | 14.94 | 72 | 12.05 | 28 |
|  | HiBT | ITO/TiO_2_/CsPbI_3_/HiBT/MoO_x_/Ag | 1.21 | 20.35 | 83.70 | 20.58 | This work |

**Table S5**. The values of decay amplitude constants and decay time.

| Films | *A*_1_ | *τ*_1_ (ns) | *A*_2_ | *τ*_2_ (ns) | *τ*_ave_ (ns) |
| --- | --- | --- | --- | --- | --- |
| CsPbI_3_ | 0.59 | 82.37 | 1.58 | 3.96 | 73.43 |
| CsPbI_3_/HBT | 0.43 | 37.69 | 0.51 | 5.29 | 33.01 |
| CsPbI_3_/HiBT | 0.51 | 30.55 | 0.48 | 3.42 | 27.96 |

**References**

1. T. Lin, Y. Hai, Y. Luo, L. Feng, T. Jia, J. Wu, R. Ma, T. A. Dela Peña, Y. Li, Z. Xing, M. Li, M. Wang, B. Xiao, K. S. Wong, S. Liu, G. Li, *Adv. Mater.* **2024**, *36*, 2312311.
2. Gaussian 16, Revision C.02, M. J. Frisch, G. W. Trucks, H. B. Schlegel, G. E. Scuseria, M. A. Robb, J. R. Cheeseman, G. Scalmani, V. Barone, G. A. Petersson, H. Nakatsuji, X. Li, M. Caricato, A. V. Marenich, J. Bloino, B. G. Janesko, R. Gomperts, B. Mennucci, H. P. Hratchian, J. V. Ortiz, A. F. Izmaylov, J. L. Sonnenberg, D. Williams-Young, F. Ding, F. Lipparini, F. Egidi, J. Goings, B. Peng, A. Petrone, T. Henderson, D. Ranasinghe, V. G. Zakrzewski, J. Gao, N. Rega, G. Zheng, W. Liang, M. Hada, M. Ehara, K. Toyota, R. Fukuda, J. Hasegawa, M. Ishida, T. Nakajima, Y. Honda, O. Kitao, H. Nakai, T. Vreven, K. Throssell, J. A. Montgomery, Jr., J. E. Peralta, F. Ogliaro, M. J. Bearpark, J. J. Heyd, E. N. Brothers, K. N. Kudin, V. N. Staroverov, T. A. Keith, R. Kobayashi, J. Normand, K. Raghavachari, A. P. Rendell, J. C. Burant, S. S. Iyengar, J. Tomasi, M. Cossi, J. M. Millam, M. Klene, C. Adamo, R. Cammi, J. W. Ochterski, R. L. Martin, K. Morokuma, O. Farkas, J. B. Foresman, and D. J. Fox, Gaussian, Inc., Wallingford CT, 2019. D. J. Fox, Gaussian 16, Revision C.02, Gaussian, Inc., Wallingford CT, (2019).

[3] A. D. Becke, *J. Chem. Phys.* **1993**, *98*, 1372-1377.

[4] S. Grimme, J. Antony, S. Ehrlich, H. Krieg, *J. Chem. Phys.* **2010**, *132, 154104*.

[5] B. P. Pritchard, D. Altarawy, B. Didier, T. D. Gibson, T. L. Windus, *J. Chem. Inf. Model.* **2019**, *59*, 4814-4820.

[6] T. Lu, F. Chen, *J. Comput. Chem.* **2012**, *33*, 580-592.

[7] W. Humphrey, A. Dalke, K. Schulten, *J. Mol. Graph.* **1996**, *14*, 33-38.

[8] Kresse, D. Joubert, *Phys. Rev. B* **1999**, *59*, 1758-1775.

[9] P. E. Blöchl, *Phys. Rev. B* **1994**, *50*, 17953-17979.

[10] V. Wang, N. Xu, J.-C. Liu, G. Tang, W.-T. Geng, Comput. Phys. Commun. 2021, 267, 108033.

[11] Z. Zhang, J. Fu, Q. Chen, J. Zhang, Z. Huang, J. Cao, W. Ji, L. Zhang, A. Wang, Y. Zhou, B. Dong, B. Song, *Small* **2023**, *19*, 2206952.

[12] J. Yuan, X. Ling, D. Yang, F. Li, S. Zhou, J. Shi, Y. Qian, J. Hu, Y. Sun, Y. Yang, X. Gao, S. Duhm, Q. Zhang, W. Ma, *Joule* **2018**, *2*, 2450-2463.

[13] Y. Ding, Q. Guo, Y. Geng, Z. Dai, Z. Wang, Z. Chen, Q. Guo, Z. Zheng, Y. Li, E. Zhou, *Nano Today* **2022**, *46*, 101586.

[14] S. Öz, A. K. Jena, A. Kulkarni, K. Mouri, T. Yokoyama, I. Takei, F. Ünlü, S. Mathur, T. Miyasaka, *ACS Energy Letters* **2020**, *5*, 1292-1299.

[15] S. S. Mali, J. V. Patil, J. A. Steele, S. R. Rondiya, N. Y. Dzade, C. K. Hong, *ACS Energy Letters* **2021**, *6*, 778-788.

[16] W. Jeong, S. R. Ha, J. W. Jang, M.-K. Jeong, M. D. W. Hussain, H. Ahn, H. Choi, I. H. Jung, *ACS Applied Materials & Interfaces* **2022**, *14*, 13400-13409.

[17] C. Duan, A. Tang, Q. Guo, W. Zhang, L. Yang, Y. Ding, Z. Dai, E. Zhou, *Adv. Funct. Mater.* **2024**, *34*, 2313462.

[18] Y. Bai, Z. Zhou, Q. Xue, C. Liu, N. Li, H. Tang, J. Zhang, X. Xia, J. Zhang, X. Lu, C. J. Brabec, F. Huang, *Adv. Mater.* **2022**, *34*, 2110587.

[19] J. Tian, Q. Xue, X. Tang, Y. Chen, N. Li, Z. Hu, T. Shi, X. Wang, F. Huang, C. J. Brabec, H.-L. Yip, Y. Cao, *Adv. Mater.* **2019**, *31*, 1901152.

[20] X. Liu, S. Fu, W. Zhang, Z. Xu, X. Li, J. Fang, Y. Zhu, *ACS Appl. Mater. Interfaces* **2021**, *13*, 52549-52559.

[21] X. Li, W. Chen, S. Wang, G. Xu, S. Liu, Y. Li, Y. Li, *Adv. Funct. Mater.* **2021**, *31*, 2010696.

[22] Q. Xiao, J. Tian, Q. Xue, J. Wang, B. Xiong, M. Han, Z. Li, Z. Zhu, H.-L. Yip, Z. a. Li, *Angew. Chem. Int. Ed.* **2019**, *58*, 17724-17730.

[23] P. Wang, H. Wang, M. Jeong, S. M. Lee, B. Du, Y. Mao, F. Ye, H. Zhang, D. Li, D. Liu, C. Yang, T. Wang, *J. Mater. Chem. C* **2020**, *8*, 8507-8514.

[24] Z. Li, J. Wang, Y. Deng, J. Xi, Y. Zhang, C. Liu, W. Guo, *Adv. Funct. Mater.* **2023**, *33*, 2214562.

[25] C. Liu, C. Igci, Y. Yang, O. A. Syzgantseva, M. A. Syzgantseva, K. Rakstys, H. Kanda, N. Shibayama, B. Ding, X. Zhang, V. Jankauskas, Y. Ding, S. Dai, P. J. Dyson, M. K. Nazeeruddin, *Angew. Chem. Int. Ed.* **2021**, *60*, 20489-20497.

[26] C. Duan, F. Zou, S. Li, Q. Zhu, J. Li, H. Chen, Z. Zhang, C. Chen, H. Guo, J. Qiu, K. Wang, Y. Dong, Y. Qiu, L. Ding, X. Lu, H. Luo, K. Yan, *Adv. Energy Mater.* **2024**, *14*, 2303997.

[27] H. Liu, Q. Tu, D. Wang, Q. Zheng, *Dyes Pigments* **2021**, *191*, 109368.

[28] D. Zhang, P. Xu, T. Wu, Y. Ou, X. Yang, A. Sun, B. Cui, H. Sun, Y. Hua, *J. Mater. Chem. A* **2019**, *7*, 5221-5226.
